# Early‐growth trajectories affect juvenile survival, age at first reproduction and lifetime fitness in a long‐lived seabird, the little penguin

**DOI:** 10.1111/1365-2656.70124

**Published:** 2025-09-01

**Authors:** Justine Wintz, Nicolas Joly, Stéphanie Jenouvrier, Vincent A. Viblanc, Andre Chiaradia, Claire Saraux

**Affiliations:** ^1^ Université de Strasbourg, CNRS, IPHC UMR7178, F‐67000 Strasbourg France; ^2^ Biology Department Woods Hole Oceanographic Institution Woods Hole Massachusetts USA; ^3^ Conservation Department Phillip Island Nature Parks Cowes Victoria Australia; ^4^ School of Biological Sciences Monash University Clayton Victoria Australia

**Keywords:** early development, early environmental effects, fitness, individual heterogeneity, individual quality, life‐history traits, silver spoon effects

## Abstract

1. Early environmental conditions experienced during juvenile growth are known to have marked effects on adult phenotypes in animal populations. Yet, the life‐history outcomes of variable growth strategies have rarely been investigated in wild populations.
2. The aim of this study was to examine the natural variation of growth patterns displayed within a seabird population and assess their impact on juvenile survival, age at first reproduction, lifetime reproductive outputs (LRO) and longevity.
3. Using a 26‐year study on the ecology of little penguins, we compiled over 2200 chick growth curves and defined 11 growth parameters classified by magnitude, form and rate. Although the growth curves formed a continuum according to these 11 growth parameters, non‐supervised statistical clustering showed that growth trajectories clustered into three main groups: fast, slow and light. Fast chicks (*n* = 48%) attained the highest maximum mass in the shortest amount of time, whereas slow chicks (*n* = 33%) stood out by a prolonged (+7 days, i.e. +13% in comparison to fast chicks) and irregular period of juvenile growth. Finally, light chicks (*n* = 19%) reached low maximum and fledging masses (~−350 g; −37% and −36% of fast and slow chicks).
4. We tested for the effects of chick growth parameters on subsequent annual vital rates estimated through capture–mark–recapture methods as well as longer term effects on life‐history outcomes using Markov chain models. Fast and slow individuals had the highest survival rates from hatching to yearling age (19% and 17%, respectively), while light chicks were at a disadvantage during this initial period (3% survival). Fast individuals reproduced 12% earlier (2.6 years old) than slow individuals, had 12.5%–88% greater longevity (up to 21 years old), and produced 1.2–3.8 times more eggs over their lifespan than slow and light individuals, respectively.
5. Fast chicks reached maturity faster and produced more offspring during their lifetime without discernible negative effects to their longevity, highlighting possible silver spoon effects.

## Graphical abstract

This study highlights the short‐ and long‐term effects of early development on life‐history outcomes in little penguins. By describing chick growth using 11 different parameters (including growth irregularity), weshowed that different growth trajectories lead to differences in long‐term fitness. In particular, chicks that grew the fastest and with the highest maximum mass exhibited the highest  reproductive success and longevity. Conversely, weak or irregular growth imposed costs in terms of individual fitness.

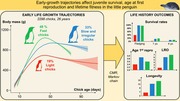

## 1 INTRODUCTION

Early development, the period from conception to maturity (Monaghan, 2008), is a critical period in the life cycle of many organisms that has influences on individual life trajectories and population dynamics (Vindenes & Langangen, 2015). Early development trajectories can vary greatly within natural populations due to variation in environmental conditions (i.e. food availability or temperature; Chappell et al., 1990; Dawson et al., 2005), parental care or quality (Sofaer et al., 2018; Wendeln & Becker, 1999), parasitism or infections (Granroth‐Wilding et al., 2014; Reed et al., 2012), the position in the family hierarchy (hatching order in birds; Song et al., 2019), or sex (Richner, 1991). These differences are particularly evident when examining among‐individual variability in juvenile growth rates (Monaghan, 2008), though determining the fitness consequences of variable growth trajectories can prove complex.

On one hand, early and rapid growth may positively affect individual fitness (Fay et al., 2018) by allowing earlier and more efficient protection against predators (Sogard, 1997), a higher capacity at buffering initially low foraging efficiency (due to higher body mass at independence; Orgeret et al., 2016), or earlier age at sexual maturity and recruitment in the breeding population (Hutchings, 1993). On the other, the energy allocated to growth trades off with other vital functions such as immunity, resistance to oxidative stress or defence against parasites, so that rapid growth trajectories may also incur substantial fitness costs (Charnov, 1993; Roff, 1992; Stier et al., 2014; Van Der Most et al., 2011).

In addition, early environmental effects can markedly affect growth patterns. Typically, individuals raised under good environmental conditions (e.g. plentiful resources, low levels of parasites, high‐quality parents) may be able to invest energy in multiple traits simultaneously, providing them with a fitness advantage retained through life (‘silver spoon effects'; Cooper & Kruuk, 2018; Van De Pol et al., 2006). In contrast, individuals encountering poor early environmental conditions during development may experience an alternance of slowed (or arrested) growth, followed by rapid growth when favourable conditions improve. These ‘catch‐up’ patterns are also thought to impose fitness costs to the organisms (Inness & Metcalfe, 2008; Metcalfe & Monaghan, 2001).

Whereas several studies have investigated the fitness outcomes of variable early‐life growth trajectories in laboratory conditions (Inness & Metcalfe, 2008; Lee et al., 2013), fewer have done so in the wild (Lemaître et al., 2015). This is partly because evaluating the net impact of early growth on individual fitness in wild populations requires: (1) evaluating the existence of alternative growth trajectories within given populations; and (2) evaluating the consequences of trajectories on multiple, interconnected, life‐history outcomes concurrently (Stearns, 1989; Veylit et al., 2020). The latter requires longitudinal monitoring of individuals from birth to death in order to evaluate the effects of alternative growth trajectories on key vital rates such as age‐specific survival or fecundity.

Here, using a unique long‐term monitoring study (26 years) on the ecology and life‐history of little penguins (*Eudyptula minor*), we tested the influences of early‐life growth trajectories on individual life‐history outcomes. First, we analysed over 2200 little penguin chicks' growth curves in terms of magnitude, form and rate (Ricklefs, 1973) to assess the breadth of growth trajectory variation present in the population. While there is a continuous range of growth trajectories in our population, we used an unsupervised clustering approach to identify growth trajectories categories across the population, subsequently allowing us to test for differences in the fitness outcomes of those mean trajectories. Second, we tested the effects these mean growth trajectories had on individual vital rates, including juvenile and adult survival rates, the probability to reproduce and the probability to produce a second clutch within each season. Finally, we used these vital rates to explore the links between early growth and individual life‐history outcomes, including age at first reproduction, lifetime reproductive outputs and longevity.

With a potential lifespan of up to 25 years (Dann et al., 2005), little penguins are expected to favour somatic over reproductive investments when food availability decreases (Hirshfield & Tinkle, 1975; Lack, 1968; Williams, 1966). As a result, any small deterioration in environmental conditions should be rapidly defrayed on the offspring, resulting in an important variability in offspring early developmental conditions and growth rates (Jenouvrier et al., 2015). Due to a highly asynchronous breeding season spanning up to 5 months (Joly et al., 2022; Reilly & Cullen, 1981), chicks are exposed to highly variable environmental conditions both within and between breeding seasons (Pelletier et al., 2012). This is further amplified by strong foraging constraints due to one of the smallest foraging ranges among seabirds when breeding (some 20 km from the breeding colony; Chiaradia et al., 2007; Collins et al., 1999). Thus, in this species, the guard duration (period during which both parents alternate between parental care on‐land and hunting at sea) is a key parameter reflecting the quality of environmental conditions during growth (Chiaradia & Nisbet, 2006), with better parental foraging conditions leading to an extended and longer guard period (Saraux et al., 2011).

We hypothesized that different growth trajectories should lead to different fitness outcomes. On the short term, we expected chicks with high masses at independence to exhibit higher fledging success and juvenile survival (Fay et al., 2018; Sogard, 1997), and chicks with rapid growth to reproduce earlier (lower age of first reproduction). Faster early growth is often associated with earlier reproductive onset, as individuals that reach critical body condition or size thresholds sooner are physiologically capable of reproducing at a younger age (Hutchings, 1993). This pattern is consistent with life‐history theory, which predicts that individuals on faster growth trajectories may adopt earlier maturation strategies, especially in variable or high‐mortality environments (Hutchings, 1993). Over the longer term, we expected chicks raised in favourable environmental conditions (prolonged guard duration) and showing a regular growth pattern leading to high peak body mass and fledging mass to retain an advantage throughout their lives ('silver spoon effect'; Cooper & Kruuk, 2018; Van De Pol et al., 2006). Specifically, we expected chicks raised in favourable environmental conditions to exhibit longer lifespans and produce more offspring (Cooper & Kruuk, 2018; Van De Pol et al., 2006). In contrast, we expected irregular growth trajectories to impose somatic and fitness costs similar to those often found for organisms displaying compensatory growth patterns (Hector & Nakagawa, 2012). Testing the links between early growth and multiple life‐history outcomes, including age at first reproduction, lifetime reproductive outputs and longevity, was expected to provide initial indications on whether specific mean growth trajectories may be favoured by selection in wild populations.

## 2 MATERIALS AND METHODS

### 2.1 Study site and bird monitoring

The studied colony is located in the Summerland Peninsula on Phillip Island (38°30′38.62″ S, 145°8′58.76″ E), Victoria, Australia, and hosts 28,000–32,000 breeding adults (Sutherland & Dann, 2014). For this study, 118 wooden artificial nest boxes were used in the Penguin Parade® area with an annual occupation rate ranging between 31% and 66%. The nest boxes were surveyed three times a week during the breeding season, from September to March over 26 years (1995–1996 and 2000–2023). As breeding spans two calendar years, we refer to a breeding season as, for example, 2001 for the 2001–2002 breeding season. From laying, the little penguin's breeding cycle is divided into three stages: incubation, chick guard (when one parent stays with the chicks), and post‐guard (when chicks are left alone during the day and parents only return at night to feed them; see Chiaradia & Kerry, 1999; Reilly & Cullen, 1981). To minimize birds' disturbance, chicks were weighed only in the absence of parents, during the post‐guard period.

#### 2.1.1 Chick monitoring

Retaining only chicks weighed at least five times over their growth period resulted in 2298 growth curves included in this study (see the Appendix S2). Within a clutch, little penguins produce two eggs that generally hatch within 24 h of each other. Thus, chick age (number of days post‐hatching) was known within ±1 day. From the first day of post‐guard until they died or fledged, chicks were weighed using either an electronic kitchen scale to the nearest 1 g or a Pesola spring scale to the nearest 10 g depending on the year. Chicks were individually marked at the first weighing using food colouring dye on the lower breast feathers, and this was repeated as soon as the dye began to fade. At the age of ~7 weeks, chicks heavier than 700 g were marked by implanting an electronic transponder (Allflex, Capalaba, Australia) subcutaneously between the shoulder blades (Chiaradia & Kerry, 1999), allowing for individual identification throughout their lifetime. Chicks were considered to have fledged when they left the nest older than 45 days and had reached a peak body mass ≥800 g (see Chiaradia & Nisbet, 2006).

#### 2.1.2 Adult monitoring

Adults were monitored using an Automated Penguin Monitoring System (APMS) (Chiaradia & Kerry, 1999), and a manual transponder reader. The APMS consists of two sets of RFID antennas located on the main routes taken by the birds when leaving to sea or returning to the colony. This system automatically records birds' ID (PIT tag number), the date and time of passage. The manual transponder reader was used to identify individuals present in a nest during surveys conducted three times a week to determine breeding status. The recapture probability of this monitoring was estimated at 0.76 for 1‐year‐old individuals and >0.9 for older individuals (see capture–mark–recapture model; Appendix S3D). The monitoring of nests further informed us about which individuals bred, the number of clutches they produced per season (1–2, exceptionally 3; Reilly & Cullen, 1981), and the number of fledged offspring produced per clutch (0–2).

### 2.2 Early growth analyses

**FIGURE 1:**
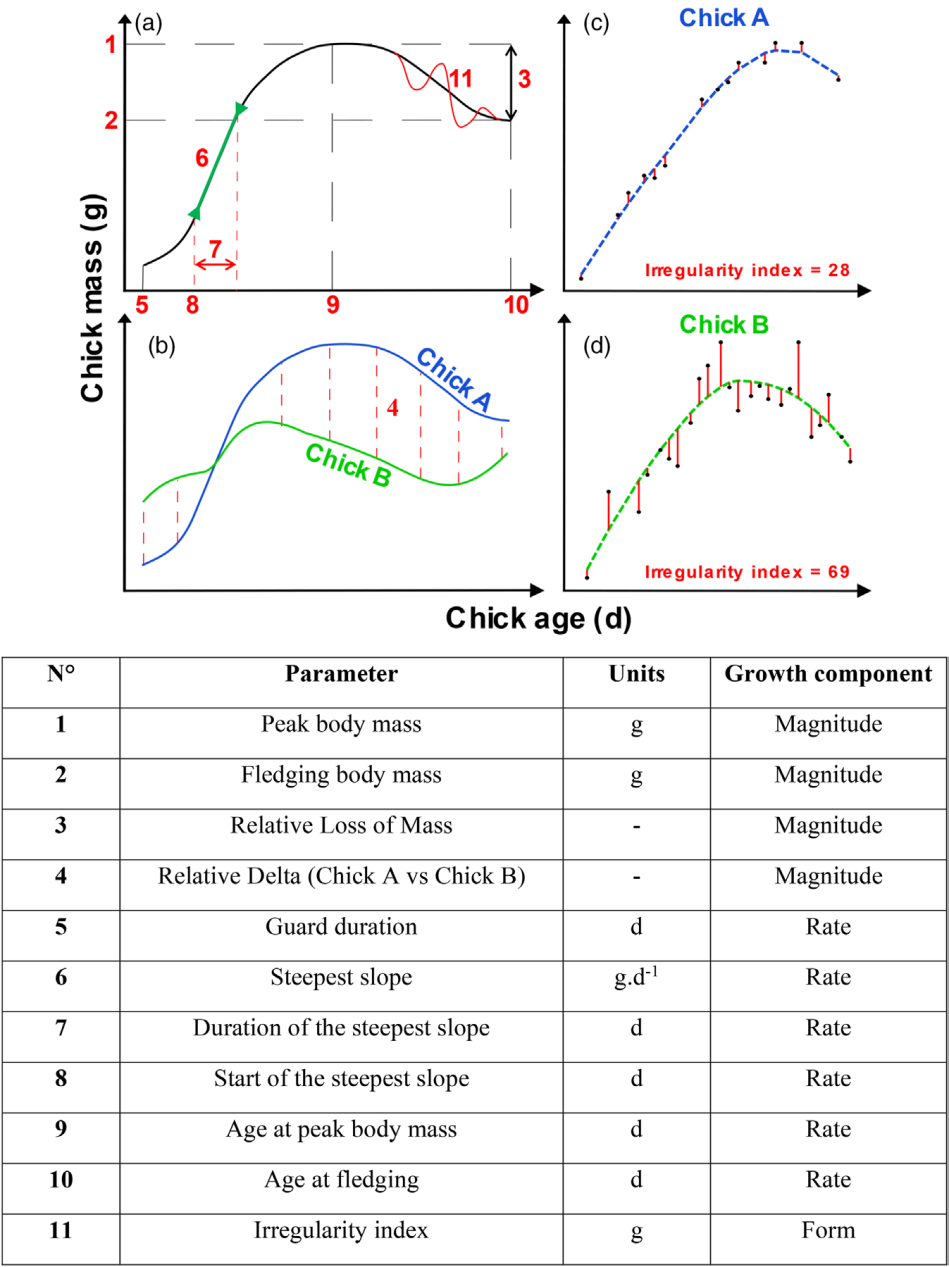
Description of the 11 growth parameters estimated: (a) Graphical representation of 10 of them; (b) Graphical representation of the relative delta (Chick A vs. Chick B, parameter 4); (c) and (d) graphical representation of two contrasted values of the irregularity index (parameter 11): The black dots represent the measured body masses, blue and green dashed lines represent the loess regression (span = 0.9) and the solid red lines indicate residuals, that are then averaged (in absoulte values) to estimate the ireegularity index. Table listing all 11 parameters along woth their units.

All analyses and statistics were done in R v 4.3.1 (R Core Team, 2023). Results are presented as mean ± SD. Chicks' growth curves were estimated through mass changes over time. Based on the literature (Chiaradia & Nisbet, 2006; Ricklefs, 1973), we used 11 parameters to characterize the three components of the growth curves: including four magnitude parameters, six rate parameters and one form parameter (see Figure 1).

#### 2.2.1 Magnitude parameters

The magnitude parameters focused on the net mass gain of the chick during development (Ricklefs, 1973) and included (1) peak body mass; (2) body mass at fledging; (3) relative mass loss between peak mass and the last mass measurement, calculated as $\text{mass loss}=\frac{\text{peak mass}-\text{last mass measurement}}{\text{peak mass}}$, and; (4) the relative growth difference $\left(d\right)$ between sibling chicks, calculated as the relative mass difference at each measurement: $d=\pm \frac{\sum_{i=1}^{N}\frac{\text{mass chick}\;A_{i}-\text{mass chick}\;B_{i}}{\text{mean}\left(\text{mass chick}\;B_{i};\text{mass chick}\;A_{i}\right)}}{N}$, where *N* is the number of measurements at the same date between chick A and chick B.

#### 2.2.2 Rate parameters

The rate parameters considered the steepness of the linear part of the growth curve and chick age at different times. These included the following: (5) guard duration, deduced from the age of the first mass measurement; (6) the steepest slope of the growth curve; (7) the duration of the steepest slope; and (8) its point of onset (start). These were estimated for each individual by extracting the segment of highest derivative (see Appendix S1A). Finally, we defined (9) age at peak body mass; and (10) age at fledging as further indicators of the rate of chick development.

#### 2.2.3 Form parameter: irregularity index

To describe the overall form of the curve, we developed an irregularity index (11), representing how much the realised growth curve deviated from its smoothed counterpart (see Figure 1; Appendix S1B).

#### 2.2.4 Principal components analysis and clustering

Given that the above variables may describe similar biological processes, we ran a principal component analysis (PCA) on the 2298 growth curves to sum up the information on growth patterns. As some variables were highly correlated (>0.5 see Cohen, 1988, 1992), we reduced the number of parameters to keep only moderate correlations (<0.5) in the PCA: a total of seven parameters including peak body mass, relative loss of mass, relative growth difference (delta) between two sibling chicks, guard duration, steepest slope, age at peak body mass and the irregularity index; see Appendix S2. Data were standardized and missing values (2.2% of total values, in particular the delta of growth between chick A and chick B for chicks that grew without a sibling) were rebuilt using the function imputePCA (package ‘missMDA’, see Josse & Husson, 2012a for more details).

To test for the existence of different mean growth trajectories in the population, we ran a clustering analysis using the unsupervised hierarchical clustering on principal components (HCPC) method on the first PCA axes (number of axes retained assessed using the expectation‐maximization method; *estim_ncpPCA* function from ‘missMDA’ R‐library v.1.18; see Josse & Husson, 2012b and Euclidean distances were used to build the hierarchical tree) with the R package FactoMineR (Husson et al., 2017). The number of clusters was determined automatically using the higher relative loss of within‐cluster inertia. We further ran a sensitivity analysis (see Appendix S2) to ensure that the number of clusters detected was robust and representative of biologically meaningful mean growth trajectories in the population. Growth parameters were then compared between clusters using an ANOVA followed by Tukey–Kramer post‐hoc tests.

### 2.3 Effects of growth trajectories on adult life‐history traits

#### 2.3.1 The little penguin life cycle

**FIGURE 2:**
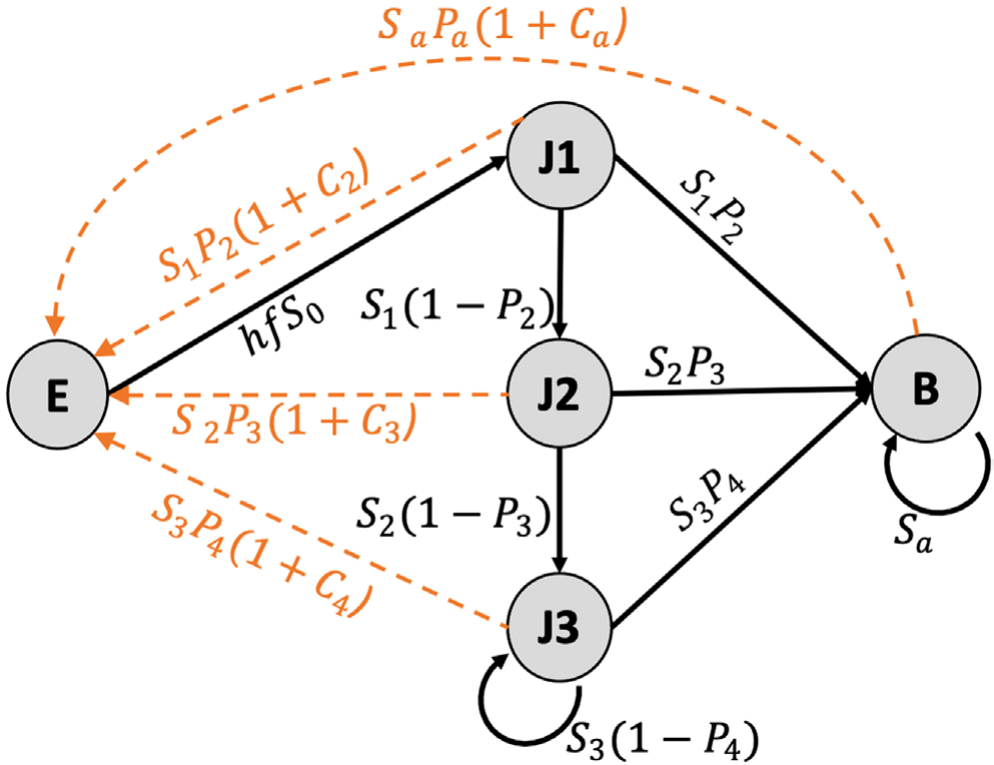
Little penguin life cycle showing the five states (circles) and the possible transitions between them (arrows) at a projection interval of 1 year. States: E = Eggs, J1 = 1‐year‐old juveniles, J2 = 2‐year‐old juveniles, J3 = 3‐year‐old juveniles, B = breeders. Solid black lines indicate transitions among surviving individuals, while orange dashed lines indicate reproduction. Probabilities of transition and reproduction are calculated with vital rates: *S*_*i*_ = survival rates at age *i*, *P*_*i*_ = Probability of first reproduction at age *i* or adult reproduction probability for *P_a_*, *f* = fledging success, *C* = second clutch probability, *h* = hatching success (from egg to post‐guard).

To understand how chick growth trajectories influenced life‐history outcomes including lifetime reproduction and longevity, we built a stage‐classified post‐breeding life cycle (Caswell, 2001; Van Daalen & Caswell, 2017), including five states (s=5): eggs (E), juveniles (J1, J2, J3) and breeders (B) (see Figure 2). Juveniles were separated into three ages to account for different ages at first reproduction (at 2, 3 or 4 years old; Joly et al., 2023) and survival rates (increased survival with age until 3 years old; Sidhu et al., 2007). Juveniles transition to the breeder stage upon first reproduction and remain in that state as long as they survive. For example, an individual that breeds at age 2 transitions directly from J1 to the breeder state, bypassing J2 and J3. We assumed breeding events occur regularly, as 94.1% of little penguins breed annually in our population (Nisbet & Dann, 2009). A few individuals lived to old ages but were never observed in our long‐term breeding monitoring. Those individuals were discarded from the calculation of the age at first reproduction, as they probably used non‐monitored nests.

Regarding vital rates associated with the little penguin life cycle (Figure 2), hatching success (from egg to post‐guard; h) and sex ratio (sr) could not be differentiated according to growth clusters as a cluster could only be attributed once the chick reaches post‐guard and chicks could not be sexed through anatomical features. Therefore, h was estimated from all reproduction events monitored in the colony over the 25 years and sr was assumed even (sr = 0.5). At each breeding event, two eggs can be produced, therefore the term 2 × sr = 2 × 0.5 = 1 can be simplified in the life cycle at each reproduction event. As the probability of detecting a juvenile at the colony after the fledging is low (penguins do not automatically return to their natal colony before breeding), a capture‐mark‐recapture model (CMR; Lebreton et al., 1992) was used to estimate survival rates ($S_{0},S_{1},S_{2},S_{3},S_{a}$). The construction and choice of the CMR model are detailed in Appendix S3. Fledging success (f) was calculated using a GLMM with a Binomial distribution and included mother and father IDs as random effects: GLMM: fledging success ~ growth cluster + (1|ID mother) + (1|ID father). Probabilities of first reproduction at different ages ($P_{2},P_{3},P_{4},P_{a}$) were calculated using a GLM with a Binomial distribution. Here, the parents' identities were not used as random effects as too few chicks reproduced compared to the number of random factor groups (76 breeder individuals, for 56 mothers and 58 fathers). In our longitudinal database, third clutches occurred only twice out of 541 breeding events. We therefore considered these events to be negligible, and only calculated the probability of producing a second clutch. Second clutch probability (C; producing up to four eggs in a season) was calculated using a GLMM with a Binomial distribution and included individual ID as a random effect: GLMM: Second clutch probability ~ growth cluster + (1|ID individual). These three models were followed by a Tukey–Kramer post‐hoc test to test for differences between growth clusters. Probability values were given in relation to the IC 95% given by the Tukey test.

#### 2.3.2 A Markov chain formulation of the life cycle

The life cycle of the little penguin (Figure 2) defines the transition structure of a finite‐state Markov chain with death as an absorbing state (Caswell, 2001, 2009; Jenouvrier et al., 2018; Van Daalen & Caswell, 2017). The matrices used to build the Markov chain model are detailed in Appendix S4.

##### Longevity and pace of life

The Markov chain model enables the calculation of various demographic outputs that are a function of an individual's initial state and its pathway through the life cycle (Roth & Caswell, 2018). The longevity of an individual was deduced from the fundamental matrix **N** (sum of each column, see Appendix S4; Caswell, 2009). The mean occupancy time in a target set (e.g. the juvenile state) was the sum of the mean occupancy times in each target state (e.g. J1, J2, J3 states) deduced from the fundamental matrix **N**. The distribution of occupancy times provides information on different life histories in the population. The probability to reproduce is the probability of reaching the breeder target state conditional on starting in state i. The mean time needed to reach the breeder state was also calculated to identify different paces of life and notably the mean age at first reproduction (Roth & Caswell, 2018).

##### Lifetime reproductive outputs (LRO), Markov chain with rewards

Reproductive outputs, or rewards, were associated with each transition in the P matrix (including the transition of remaining in a state; Van Daalen & Caswell, 2017). Individuals accumulated rewards (e.g. eggs) as soon as they were in the breeder state. This measure reflects both longevity and the pace and timing of reproduction for a given phenotype.

## 3 RESULTS

### 3.1 Early growth characterization

#### 3.1.1 Principal components analysis

**FIGURE 3:**
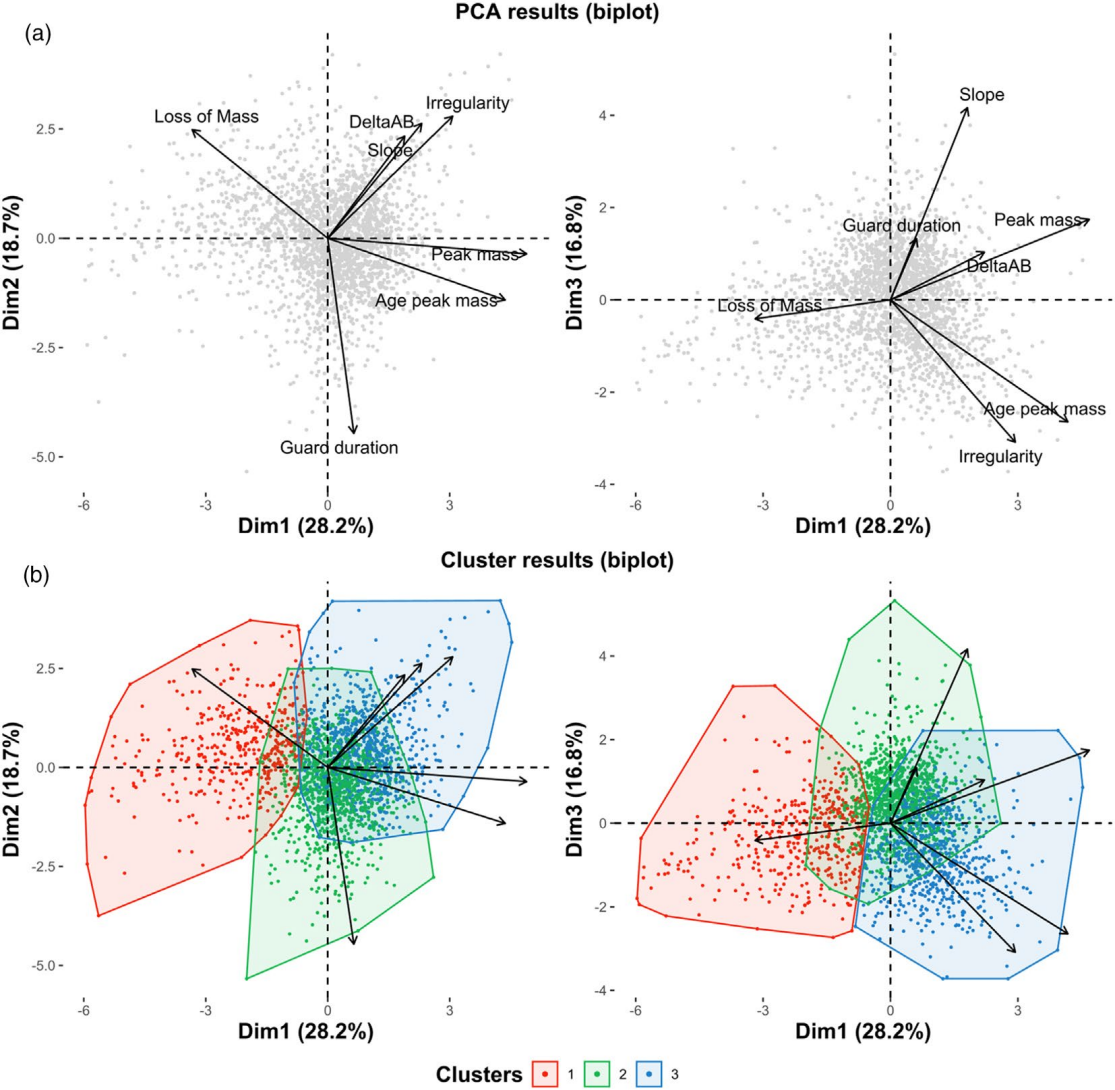
Principal component analysis (PCA) and clustering performed on the seven growth parameters describing the 2298 growth curves. (a) Projection on the three first axes of the PCA results. Grey points represent individual chick growth trajectories, while the arrows correspond to the projection of the seven growth parameters. (b) Projection on the three first axes of the clustering results. Colour points represent individual chick growth trajectories, depending on the cluster number (1 = light, 2 = fast, 3 = slow), minimum convex polygons are represented in transparency, while the arrows correspond to the projection of the seven growth parameters.

The first three principal components of the PCA explained 63.7% of the total variance (28.2%, 18.7% and 16.8%, respectively). The first axis contrasted chicks according to their growth magnitude; the second mainly classified individuals according to their guard duration, while the third highlighted the opposition between slow and irregular growth rates (negative PC3) versus high steepest slopes (positive PC3) (Figure 3a).

#### 3.1.2 Defining growth trajectories: Three types of growth

We identified three clusters of growth trajectories in our data (HCPC clustering; Figure 3b), with the magnitude, rate and irregularity parameters of the growth curves differing between clusters (see Appendix S5). A sensitivity analysis suggested that the PCA and cluster results were robust (see Appendix S2), highlighting differences in mean growth trajectories within our little penguin population.

**FIGURE 4:**
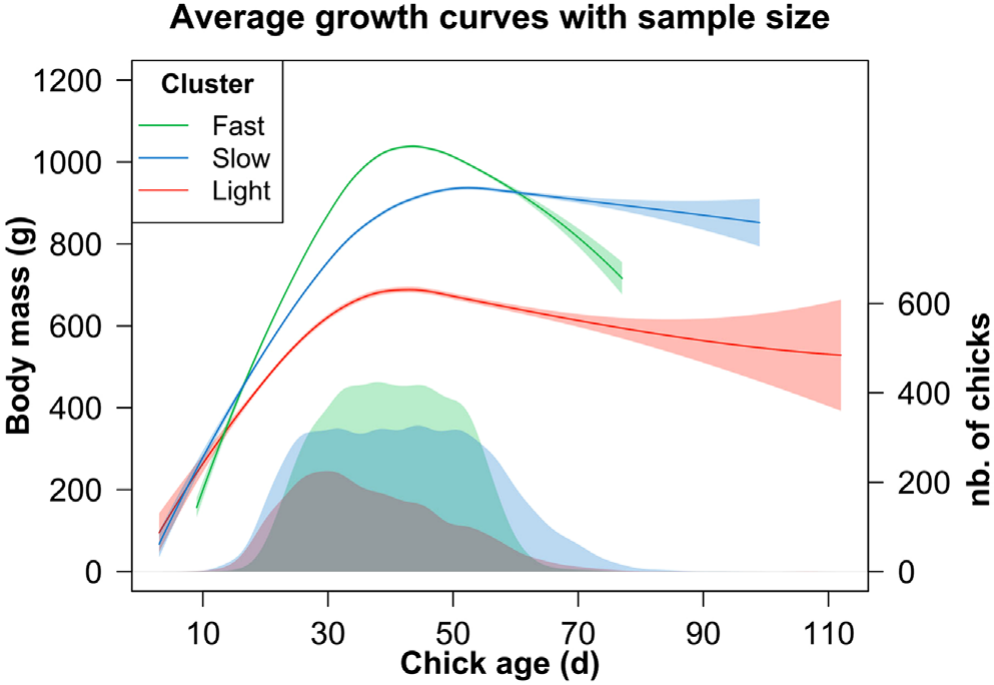
Average growth curves per cluster. Curves were smoothed with the loess function (span = 0.7) with 95% confidence interval calculation. As the number of individuals sampled at each time point varies due to chicks dying or fledging, this number is indicated below the curves using a Gaussian kernel density (smoothing parameter fixed at 0.2) scaled back to the total number of individuals (right axis).

Briefly, chicks from Cluster 1 (*n* = 436) reached overall lower masses, with negative PC1, and were consistently lighter than chicks from other clusters (by about one‐third) over their entire growth (Figures 3b and 4). They exhibited the lowest steepest slope at 18 g/day, compared to chicks from Cluster 2 (28 g/day) and Cluster 3 (25 g/day). We classified these chicks as *Light* individuals. Chicks from Cluster 2 (*n* = 1104) grew the fastest and reached the largest masses, with positive PC3. They reached peak mass the earliest and also benefited from the most prolonged guard period (6 days longer than the two other groups) (Figures 3b and 4). We classified these chicks as *Fast* individuals. Chicks from Cluster 3 (*n* = 758) reached relatively high masses, but growth dynamics were slow and irregular, with positive PC1 and negative PC3. They reached peak mass later than chicks from the other groups (+10 to 20 days), even though they exhibited high peak and fledging body masses (Figures 3b and 4). We classified these chicks as *Slow* individuals.

### 3.2 Vital rates depending on growth clusters

#### 3.2.1 Fledging success

Fledging success varied strongly according to chick growth trajectories (GLMM; Table 1). *Fast* and *slow* individuals had a fledging success 2.5 times higher than *light* chicks.

**TABLE 1:** Vital rates of the three growth clusters.

	Growth cluster		Fast			Slow			Light			*p*‐value		All clusters
			Mean	95% CI	*n*; *N*	Mean	95% CI	*n*; *N*	Mean	95% CI	*n*; *N*	F vs. S; F vs. L; S vs. L	Signif.	
Vital rates	Hatching success	*h*	Estimated from all breeding records											0.796
														*n* = 3768; *N* = 648
	Sex ratio	sr	Assumed balanced											0.5
	Fledging success	*f*	0.96	[0.94;0.97]	*n* = 1104	0.98	[0.97;0.99]	*n* = 758	0.39	[0.34;0.45]	*n* = 436	0.01; 0.00; 0.00	**; ****; ****	
	Survival	*S*_0_	0.2	[0.11;0.33]	*n* = 901	0.17	[0.09;0.28]	*n* = 610	0.08	[0.04;0.18]	*n* = 111			
		*S*_1_	0.7	[0.55;0.82]		0.66	[0.50;0.79]		0.47	[0.28;0.67]		Capture–mark–recapture survival model includes age, growth cluster and cohort effects		
		*S*_2_	0.83	[0.69;0.92]		0.8	[0.65;0.91]		0.65	[0.42;0.83]				
		*S*_3_	0.78	[0.64;0.83]		0.91	[0.73;0.98]		0.82	[0.54;0.95]				
		*S*_*a*_	0.9	[0.77;0.96]		0.88	[0.75;0.95]		0.77	[0.55;0.91]				
	First reproduction	*P*_2_	0.47	[0.34;0.61]	*n* = 49	0.29	[0.15;0.50]	*n* = 24	0.67	[0.15;0.96]	*n* = 3	0.32; 0.78; 0.43	ns; ns; ns	
		*P*_3_	0.5	[0.32;0.68]	*n* = 26	0.53	[0.30;0.74]	*n* = 17	0	[0.00;1.00]	*n* = 1	0.98; 0.61; 0.58	ns; ns; ns	
		*P*_4_	1	—	*n* = 13	1	—	*n* = 8	1	—	*n* = 1	—	—	
		*P*_*a*_	*No skipped breeding events assumed*											1
	Second clutch	*C*	0.32	[0.27;0.39]	*n* = 284; *N* = 49	0.3	[0.22;0.40]	*n* = 127; *N* = 24	0.12	[0.02;0.54]	*n* = 8; *N* = 3	0.91; 0.49; 0.55	ns; ns; ns	

*Note*: The vital rates of all individuals are given only when the calculation has not been done for the three clusters separately. *n* is the number of events, and *N* is the number of individuals (if different from *n*). For the first reproduction probabilities, second clutch probabilities and fledging success, GLM and GLMM were fitted for each group, followed by the Tukey post‐hoc test.Significance codes: 0.0001****, 0.01**, > 0.05 ns. For the survival rates derived from the CMR model, the 95% CI is given by the Hessian matrix.

#### 3.2.2 Survival rates from fledging

Both age and growth clusters were retained in the CMR model selection (see Appendix S3C), indicating that survival differed significantly between the three growth groups (Table 1). *Fast* chicks had the highest survival rates (from *S*_0_ = 20% to *S*_*a*_ = 90%) and *light* chicks the lowest (from *S*_0_ = 8% to *S*_*a*_ = 77%). Survival from 0 (the moment of fledging) to 1 year old was the most variable between groups: *fast* chicks had the highest survival (1.2 and 2.5 times higher than *slow* and *light* chicks respectively). For *slow* and *light* chicks, survival increased up to 3 years old, then plateaued and decreased slightly in adulthood (from *S*_0_ = 17% to *S*_3_ = 91% then *S*_*a*_ = 88% for *slow* chicks). *Fast* chicks, on the contrary, had a survival rate that decreased at 3 years old (*S*_3_ = 78%) and increased again to peak at adulthood (*S*_*a*_ = 90%).

#### 3.2.3 Age at first reproduction and second clutches

On average, the probability to first reproduce at age 2 was 29% for *slow* chicks, compared to 47% and 67% for *fast* and *light* chicks, respectively (Table 1). Note that the proportion for *light* chicks relies only on 3 birds that reached age 2, limiting the interpretation of this result. *Light* chicks also appeared to produce fewer second clutches as adults (not significantly, Table 1) than the two other groups (12% vs. 32% and 30% for the *light*, *fast* and *slow* individuals, respectively).

### 3.3 Consequences of growth heterogeneity on life‐history outcomes

**FIGURE 5:**
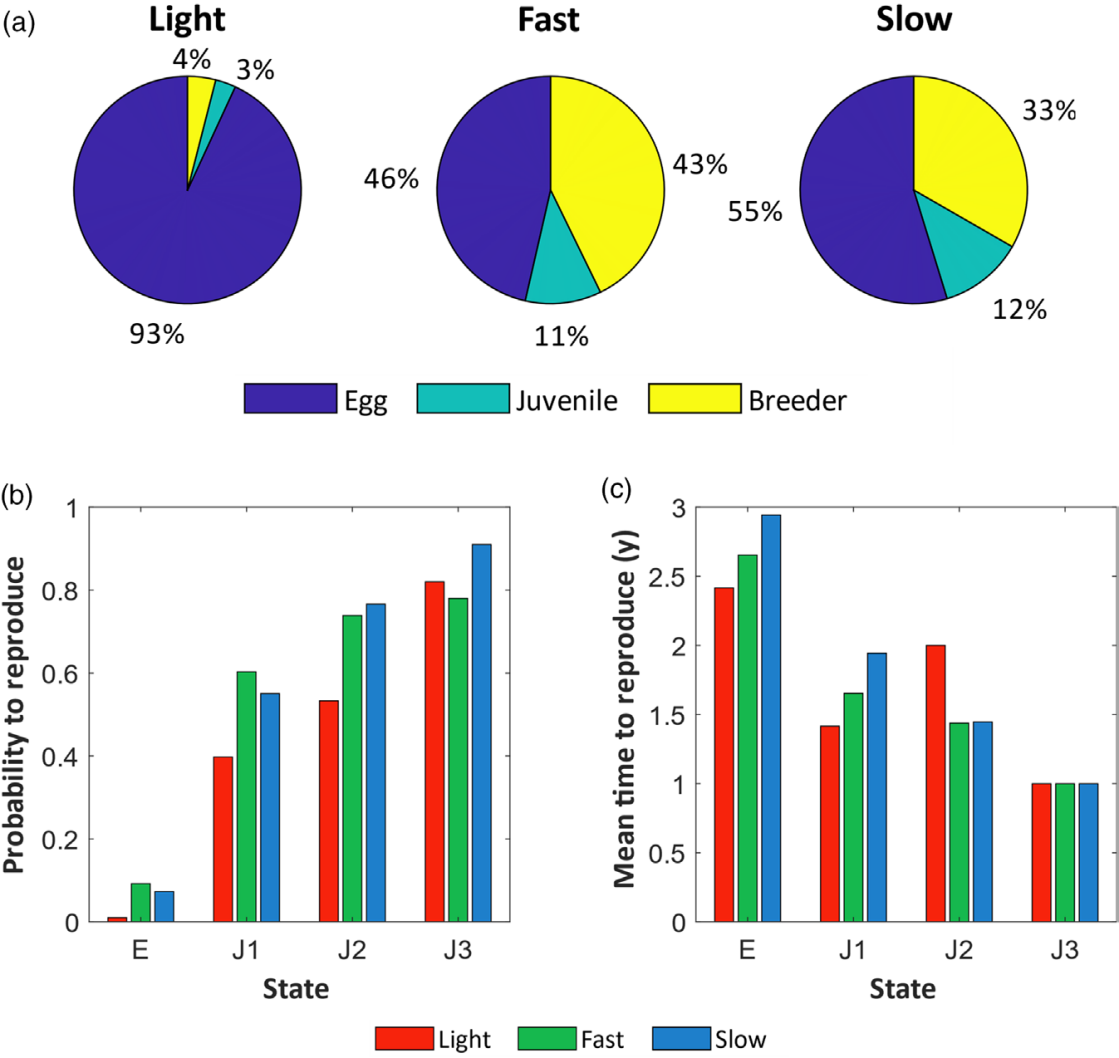
(a) State occupancy deduced from the fundamental matrices N for the three growth groups (*fast*, *slow* and *light*, see Appendix S6). Percentages represent the average time spent in each state (egg, juvenile, breeder) during the entire lifetime for individuals in each growth group. (b) Probability to reproduce for a *fast/slow/light* individual, conditional on starting in state egg (E) or juvenile (J1, J2 or J3). (c) Mean time needed to reproduce for a *fast/slow/light* individual, conditional on starting in state egg (E) or juvenile (J1, J2 or J3).

*Fast* individuals spent a higher proportion of their lifetime as breeders compared to *slow* and *light* individuals (43%, 33% and 4% for *fast*, *slow* and *light*, respectively, Figure 5a). The transition from juvenile to breeding age showed different patterns between the three groups (Figure 5b). Starting from the eggs as an initial state, *fast* chicks were more likely to recruit in the breeding population, especially compared to *light* chicks (10 times more). Although not statistically significant, *slow* chicks tended to mature later (mean age at first reproduction: 2.9 *vs*. 2.6 and 2.4 years for *slow*, *fast*, and *light* chicks, respectively; Figure 5c). These results remained similar when starting from the juvenile state J1, that is, when considering only age‐1 individuals.

#### 3.3.1 Expected longevity

**FIGURE 6:**
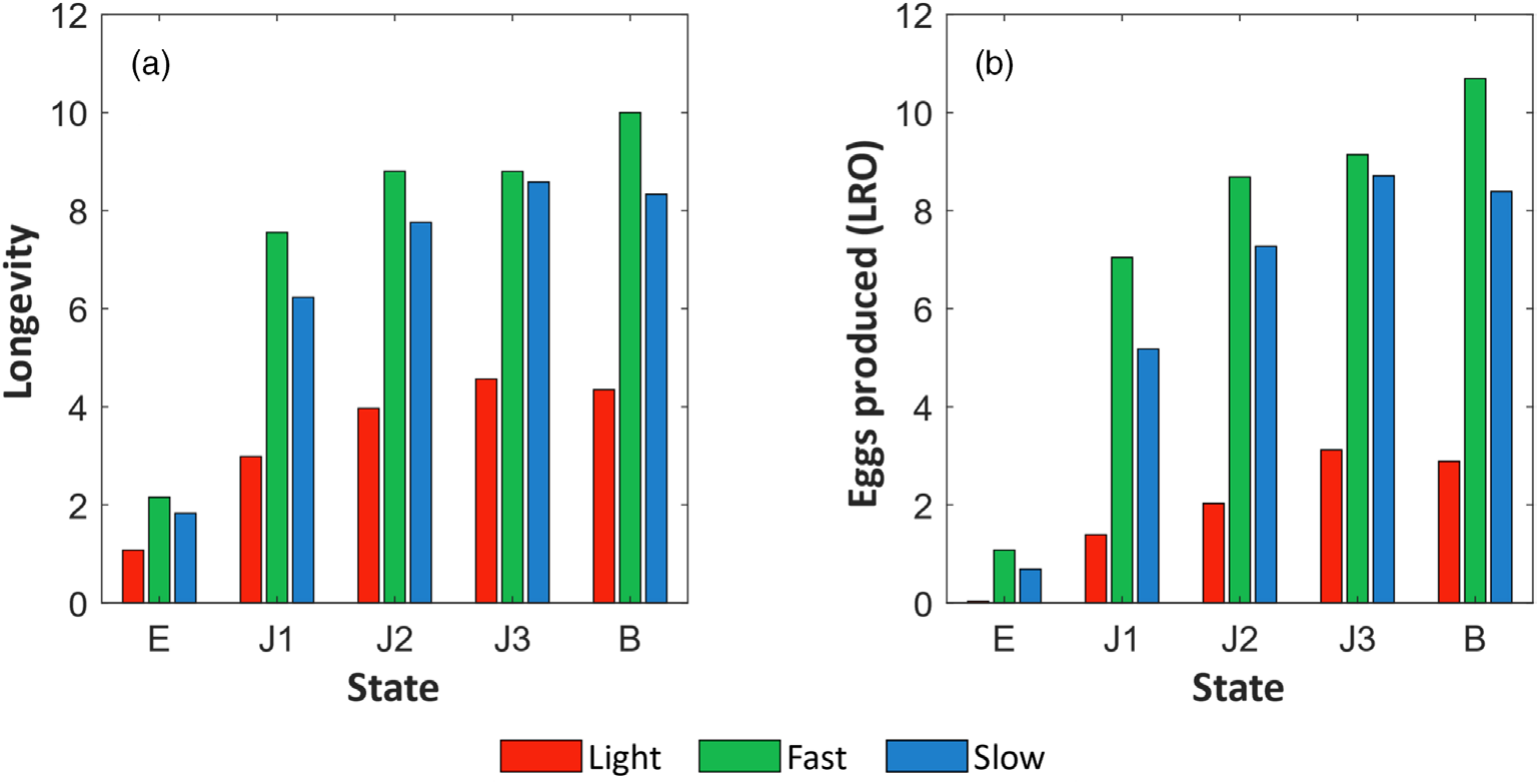
(a) Expected longevity and (b) Lifetime reproductive outputs of a *fast/slow/light* individual, conditional on starting in state egg (E), juvenile (J1, J2 or J3) or breeder (B).

Analysing data for all individuals from the egg state, *fast* individuals had the greatest expected longevity (2.2 years), followed by *slow* (1.8 years) and *light* (1.1 years) individuals (Figure 6a). These low longevities were due to low probabilities for eggs to reach the juvenile state J1 (15%, 13% and 2% for *fast*, *slow* and *light* individuals, respectively). Once individuals had passed this critical period and reached the J1 state, the remaining longevity almost tripled. Yet, differences in longevity between *fast*, *slow* and *light* individuals persisted throughout juvenile states and adulthood. Finally, the average longevity of individuals that recruited in the population was 12.6, 11.2 and 6.7 years old for the *fast*, *slow* and *light* individuals, respectively (remaining life in the breeder state + mean age at first reproduction).

#### 3.3.2 Lifetime reproductive outputs

*Fast* individuals produced the most eggs regardless of the initial state, followed by *slow* and *light* individuals (1.2 times and 3.8 times higher on average than *slow* and *light* individuals respectively; Figure 6b). For *slow* and *light* individuals, the LRO was maximal for birds in the J3 state, whereas for *fast* individuals, the LRO was maximal for birds in the breeder stage.

## 4 DISCUSSION

By combining a long‐term dataset with growth measurements and demographic modelling, we examined the impact of early growth on subsequent life‐history outcomes (Lindström, 1999). Because growth is a complex process that involves more than just speed, we described more than 2200 chick growth curves through 11 descriptors representing the magnitude, form and rate of individual growth trajectories. We developed a simple metric of growth irregularity based on repeated body mass measurements, offering a practical tool for field ecologists. Although the growth curves spanned a continuum, unsupervised clustering provided a clear and interpretable framework by consistently grouping individuals into three distinct and robust categories, as confirmed by sensitivity analyses (see Appendix S2). These clusters captured meaningful differences in growth trajectories: (1) *fast* chicks that grew fast and reached high masses; (2) *slow* chicks that showed an irregular growth pattern and required an additional 7 days to fledge at comparable high masses; and (3) *light* chicks that demonstrated consistent but weak growth patterns, peak body mass being 1.5 times less than that of *fast* chicks.

### 4.1 Short‐term impacts of growth: Fledging success, first‐year survival and age at first reproduction

Our findings underline the importance of early growth trajectories for chicks' short‐term survival; *fast* and *slow* chicks (of higher growth magnitude) showed higher fledging success and post‐fledging juvenile survival than *light* chicks. While this aligns with studies in passerines showing that heavier fledglings tend to have higher survival (Perrins, 1965; Tinbergen & Boerlijst, 1990), the relationship between fledging mass and survival is less consistent in seabirds. For example, Perrins et al. (1973) found a positive effect of mass on post‐fledging survival in Max shearwaters, but other studies in puffins (Harris & Rothery, 1985), razorbills (Lloyd, 1979), or guillemots (Hedgren, 1981) found no such relationship. In little penguins, the absence of parental care after fledging and the highly variable environmental conditions on Phillip Island over the past 25 years (Pelletier et al., 2012) might explain the important effects growth magnitude had on short‐term survival. Greater reserves to cope with a period of low foraging efficiency as juveniles (Keedwell, 2003), or an improved physical condition rendering large chicks less vulnerable to environmental fluctuations and predators in the early months (Keedwell, 2003), might have explained the advantages of reaching a high body mass at fledging.

It is hypothesized that rapid growth allows individuals to mature early, rapidly investing resources into sexual traits and reproduction (Hutchings, 1993). Therefore, one might have expected fast‐growing individuals (low age at fledging, low age at peak mass) to reproduce earlier. Our results were in line with this hypothesis, albeit with a few nuances. We found that *light* individuals were those with the earliest age at first reproduction, followed by *fast* and finally *slow* individuals. When looking at the age of peak body mass, this matches the hypothesis: the *light* ones were those who reached the peak mass the fastest, followed by the *fast* ones, then the *slow* ones. The results are more nuanced when considering the age at fledging: *light* chicks left the nest later than *fast* chicks while reproducing earlier. However, this result should be interpreted with caution, as only three individuals from the light group were observed to reproduce. Comparing the *slow* and *fast* groups revealed subtle differences in primiparity. Whereas the majority of s*low* individuals started reproducing at 3 years of age, 50% of *fast* chicks reproduced 1 year earlier (at 2 years old), only partly supporting the idea that rapid growth may lead to more advanced maturity.

Early breeding could impose an additional cost on precocious individuals, since energy demands are high during reproduction (31% of the energy budget; Gales & Green, 1990). Similar results were found for Adélie Penguin, where young breeders were at greater risk of early mortality (Ainley & DeMaster, 1980). We might therefore expect to see an increase in mortality among *fast* individuals at 3 years of age (where 50% of individuals have reproduced at 2 years) or among *slow* individuals at 4 years of age (where 53% of individuals have reproduced at 3 years). However, there was no drop in survival for either *fast* or *slow* individuals in the year following reproduction. *Fast* individuals only showed a drop in survival at 4 years, while *slow* individuals only showed an increase in survival during the juvenile phase. An experimental component would be needed to conclude on this point, but the cost of first breeding was not obvious in our data.

### 4.2 Long‐term impact of growth and silverspoon effects

Our results highlighted long‐term carry‐over effects that differed between the three clusters. *Fast* individuals grew faster, survived better and had more offspring over life. In contrast, *slow* and *light* chicks seemed to suffer the consequences of irregular or low growth, respectively, during their early development, with lower lifetime reproductive outputs and reduced longevity.

Our results support the idea that silver spoon effects (Song et al., 2019; Van De Pol et al., 2006) might underlie such lifelong differences between chicks. *Fast* individuals performed overall better in a suite of traits (i.e. higher lifetime reproductive outputs, longevity), differences which might have stemmed from more favourable early rearing conditions. Notably, *fast* individuals had the longest guard period (i.e. when parents alternate one day at sea and one day guarding the chicks), indicating a prolonged period of intense parental care, which enabled the chicks to display strong growth at the very start of their life and reach independence faster afterwards (a week earlier than slow chicks). This was likely facilitated by favourable environmental conditions (Chiaradia & Nisbet, 2006), allowing parents to extend short foraging trips before undertaking longer trips to replenish their own reserves, thereby delaying the end of chick‐guarding (Saraux et al., 2011). In contrast, *slow* and *light* chicks were probably raised under less favourable conditions, making it harder for the parents to provision them regularly or sufficiently, shortening the duration of parental care and affecting their overall condition at fledging. *Fast* individuals also benefited the most from a regular linear growth trajectory, suggesting a regular supply of food during growth, and in sufficient quantity, as this is also the group with one of the highest fledging and peak masses. On the contrary, *slow* individuals, despite reaching similar masses, exhibited slower and more irregular growth patterns, which seemed to carry survival costs over the long term. As in natural conditions growth is the composite result of a succession of periods of contrasted conditions, we suggest that growth irregularity might be an important trait to consider when evaluating the consequences of growth trajectories on life‐history outcomes.

### 4.3 Growth asymmetry between siblings reveals variation in parental and environmental effects

Comparing the growth difference of the two chicks within the same clutch (Delta AB), we found that siblings often exhibited different growth trajectories (Delta >0 for *fast* and *slow* chicks and Delta <0 for *light* chicks). To better understand the dynamics at play within a nest, we observed the proportion of pairs of chicks in each cluster (*Fast/Fast*, *Slow/Light*, *Fast/Light* etc.) (Table 2). While all types of pairs could occur, *fast* individuals were most likely to grow‐up with a fast‐growing sibling. This suggests that the parents were able to feed both chicks sufficiently and equitably to raise two *fast* chicks of optimal growth. It is likely that environmental conditions were ideal during the growth period, or that both parents were high‐quality individuals (strong investment in reproduction, high foraging qualities…). This result is in line with silver spoon effects, as described above (Cooper & Kruuk, 2018; Hamel et al., 2009; Van De Pol et al., 2006).

**TABLE 2:** Distribution of growth clusters within broods.

	Chick growth cluster		
	Fast	Slow	Light
Sibling's growth cluster			
No sibling	163 (21%)	110 (19%)	53 (15%)
Fast	343 (45%)	150 (26%)	105 (30%)
Slow	150 (20%)	194 (34%)	113 (32%)
Light	105 (14%)	113 (20%)	81 (23%)

*Note*: The table shows the number of occasions (and associated proportions) when a chick of a given growth cluster (columns) was observed with a sibling of the same or a different growth  cluster (rows) within the same brood. For example, in the *fast*‐chick group (first column), 21% were single chicks, 45% were raised with a *fast* sibling, 20% with a *slow* sibling and 14% with a *light* sibling.

Despite the high number of *fast‐fast* chicks, proportions were distributed more evenly between the other categories, suggesting that two chicks that grew‐up with the same parents and in the same environmental conditions did not always grow‐up in the same way. In particular, a *light* chick was more likely to grow with a *fast* chick (30%) or a *slow* chick (32%) than with the same type of chick (23%). *Light* chicks were therefore often paired with a sibling that exhibited a faster or more robust growth trajectory. This could reflect stronger competition between the two chicks under poor environmental conditions, when parents could favour one chick over the other in order to maximize the probability of one of them reaching fledging (Emms & Verbeek, 1991; Shizuka & Lyon, 2013), or when intrinsic differences in chick quality could play a role in determining its access to food. Several individual characteristics such as sex, hatching order or personality could indeed play a role in the access to food (Diaz‐Real et al., 2016; Richardson et al., 2019; Roulin et al., 2010; Savagian & Riehl, 2023). Parasitism could also render one chick more vulnerable and increase its energy expenditure, creating important differences within the brood (Reed et al., 2012).

Finally, *slow* chicks were more likely to grow up with a *slow* sibling, although not by far (34%). This suggests that slow growth may be a parental strategy per se. Rather than investing predominantly in one chick when conditions deteriorate (*fast*/*light* pair), they may feed both chicks simultaneously but in smaller quantities, thus producing chicks with longer growth periods, while bringing them both to fledging. Several studies show that parents can adapt their feeding strategy to increase the chances of raising the number of chicks to fledging. This is the case in prothonotary warblers (Brode et al., 2021) or in American coots (Shizuka & Lyon, 2013), where parents favour the feeding of the smallest chicks in specific conditions. These patterns suggest that chick growth trajectories within a brood are not solely determined by shared conditions, but are also shaped by asymmetric allocation, sibling competition or intrinsic differences.

## 5 CONCLUSIONS

Our findings demonstrate that early developmental trajectories are strong predictors of individual reproductive success and survival in the wild. Fast, regular, and robust growth—likely reflecting favourable environmental conditions and effective parental investment—was associated with the highest lifetime fitness outcomes. Nevertheless, individuals exhibited a range of growth trajectories, clustering into three broad types along a slow–fast continuum. Silver spoon effects probably accounted for most of the inter‐individual variability (*fast–fast* pairs of sibling chicks was the most common combination). Nevertheless, intra‐brood differences remained significant (*fast–slow*, *slow–light* or *fast–light* pairs), suggesting that other mechanisms may come into play, particularly when conditions deteriorate (i.e. genetic variation, limited resources in given periods of time, unequal parental investment, intra‐brood competition or parasitism). These results highlight the critical role of early‐life conditions in shaping life‐history outcomes and underscore the need to better understand the adaptive significance of growth variation. Further studies on the determinants of growth are thus needed to better disentangle the contribution of the environment, parental quality and investment or chick characteristics, and their interactions, to growth trajectories in the wild.

## AUTHOR CONTRIBUTIONS

Claire Saraux, Justine Wintz, Nicolas Joly, Stéphanie Jenouvrier and André Chiaradia conceived the ideas and designed methodology; André Chiaradia, field rangers, volunteers and students from the Phillip Island Nature Park collected the data; Justine Wintz, Claire Saraux and Nicolas Joly analysed the data; Justine Wintz led the writing of the manuscript; Claire Saraux, Vincent Viblanc, André Chiaradia, Stéphanie Jenouvrier and Nicolas Joly provided useful comments on the organization and the writing of the paper. All authors contributed critically to the drafts and gave final approval for publication. Our study brings together authors from a number of different countries, including scientists based in the country where the study was carried out. All authors were engaged early on with the research and study design to ensure that the diverse sets of perspectives they represent was considered from the onset. Whenever relevant, literature published by scientists from the region was cited.

## CONFLICT OF INTEREST STATEMENT

The authors declare no conflicts of interest.

## ETHICAL APPROVAL

The study was conducted with research permits issued by the Department of Environment, Land, Water and Planning, Victorian State Government, Australia, and ethics approvals from the Animal Ethics Committee of Phillip Island Nature Parks.

## Supporting information


**Appendix S1A.** Steepest slope calculation.
**Appendix S1B.** Irregularity index.
**Appendix S2.** Sensitivity analysis of the PCA and the clustering method.
**Appendix S3.** Capture mark recapture model.
**Appendix S3A.** Tests GOF.
**Appendix S3B.** Implementation of the CMR model with transience and trap‐dependence in E‐Surge.
**Appendix S3C.** Capture‐Mark‐Recapture, model selection.
**Appendix S3D.** Capture‐Mark‐Recapture, capture probabilities.
**Appendix S4.** Markov chain formulation of the life cycle.
**Appendix S5.** Growth parameters for the three clusters (comparison between the three clusters performed using the ANOVA statistical test, followed by the Tukey post‐HOC test).
**Appendix S6.** Matrixes U, F, N. U gives the probabilities of transition and survival for living individuals, F gives the fertility probabilities and N is the fundamental matrix.
**Appendix S7.** LRO in terms of fledglings.

## Data Availability

Data are available from the Figshare Repository https://doi.org/10.6084/m9.figshare.29940944.v1 (Wintz et al., 2025).
