# Supplementary material for: Early‐growth trajectories affect juvenile survival, age at first reproduction and lifetime fitness in a long‐lived seabird, the little penguin
Source: J Anim Ecol. 2025 Sep 1;94(11):2240–54. doi: 10.1111/1365-2656.70124 (PMC12586760; doi:10.1111/1365-2656.70124)
Supplement: Supplementary file 1 — Appendix S1A. Steepest slope calculation. Appendix S1B. Irregularity index. Appendix S2. Sensitivity analysis of the PCA and the clustering method. Appendix S3. Capture mark recapture model. Appendix S3A. Tests GOF. Appendix S3B. Implementation of the CMR model with transience and trap‐dependence in E‐Surge. Appendix S3C. Capture‐Mark‐Recapture, model selection. Appendix S3D. Capture‐Mark‐Recapture, capture probabilities. Appendix S4. Markov chain formulation of the life cycle. Appendix S5. Growth parameters for the three clusters (comparison between the three clusters performed using the ANOVA statistical test, followed by the Tukey post‐HOC test). Appendix S6. Matrixes U, F, N. U gives the probabilities of transition and survival for living individuals, F gives the fertility probabilities and N is the fundamental matrix. Appendix S7. LRO in terms of fledglings. [file JANE-94-2240-s001.pdf]

Supplementary material

Appendix 1A: Steepest slope calculation. A: row data (black points) and smoothed curve (dotted-blue line); B: derivative function, with minimum (red) and maximum (green) derivative segments; C: smoothed curve (dotted-blue line), location of the steepest slope (between green and red bars) and steepest slope (red-dotted line)

1) the curve was smoothed using a loess trend, with a span at 0.8;
2) the derivative was then computed using the function *diff* (package 'base' version 4.2.0), divided by the time between two measurements;
3) segments of different mean derivative using piecewise regression analyses (*breakpoints* function, package 'strucchange' version 1.5-3);
4) the section of the curve with the largest positive derivative (fastest growth) was identified as the "steepest slope".

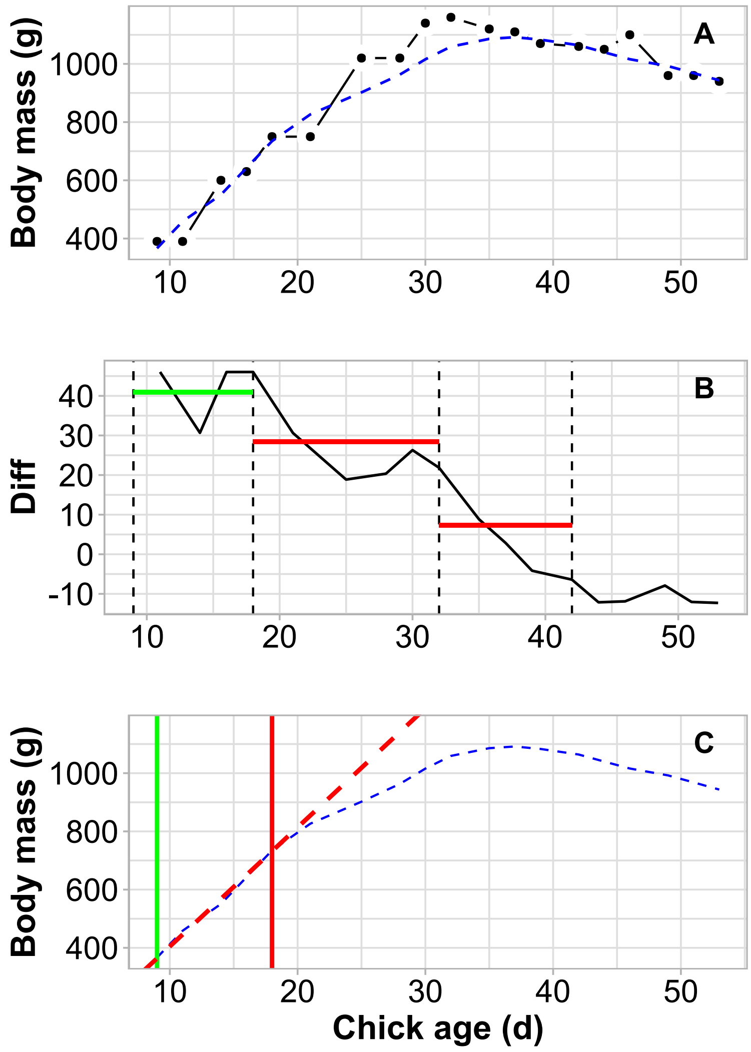

Appendix 1B: Irregularity index

We compared the actual broken stick shape of the growth curve to its smoothed counterpart (blue dotted line) describing the general shape of an individual's growth, but not all the small departures to this curve. To do so, each individual curve was smoothed using a loess (Locally Estimated Scatterplot Smoothing; package 'stats' version 4.2.0) with an average span (0.9; Simonoff, 1996). The irregularity index was then defined as the mean absolute values of the deviations from this curve (red segments).

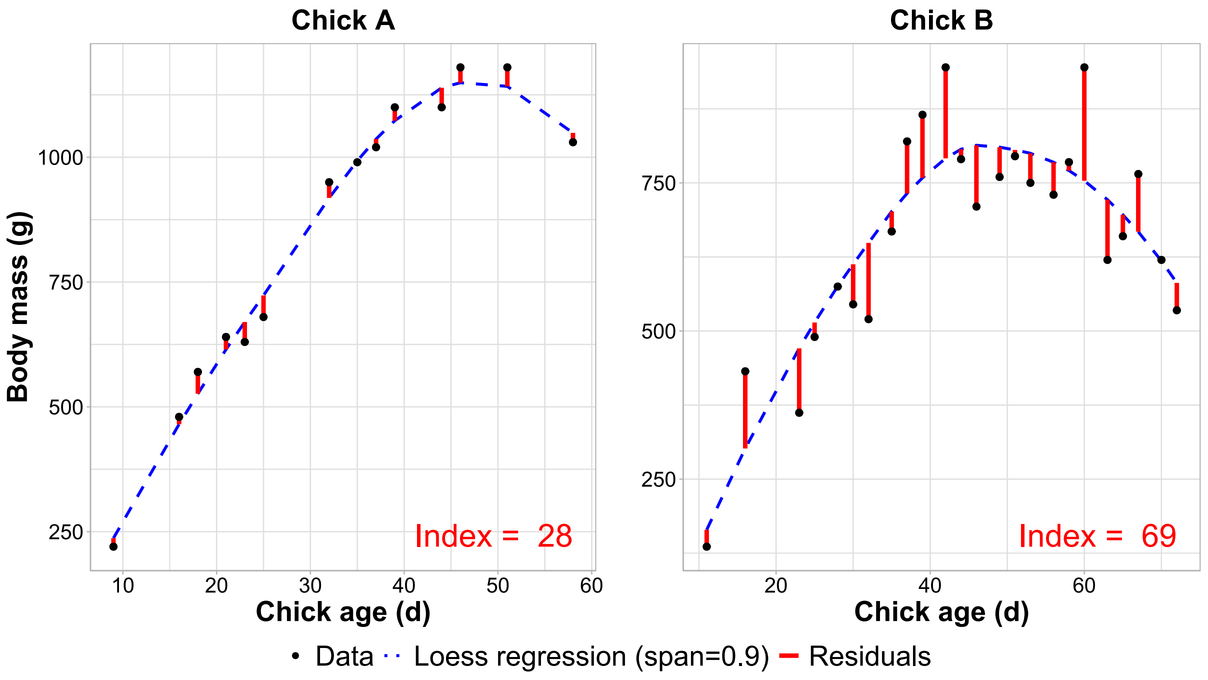

Appendix 2: Sensitivity analysis of the PCA and the clustering method

To ensure the robustness of our results, we conducted a sensitivity analysis of the growth PCA and clustering results. Here are the 3 points addressed: minimal number of points constituting a growth curve, the clustering method, and the PCA choices (variables to keep in the analysis, imputed values).

1. Minimal number of points constituting a growth curve

The first issue concerns the bias we may introduce when selecting the growth curves to keep in this study.

While decreasing the number of points necessary to build a growth curve increases the number of individuals in our analysis (+146, i.e. 6% when going from 5 to 3 for instance), it also means more uncertainty on the data, as estimating the maximum linear slope on 3 or 4 points for instance seems more uncertain. In order to find the right trade-off, we also considered the potential loss of individuals later seen as breeding that help estimating the vital rates of the different categories. To answer this question, we carried out the analysis on growth curves built from at least 3, 4, 5, 6 or 6+ points and checked whether this affected the results of the PCA and clustering.

Going from 7 to 5 points enabled us to add 3 more individuals later seen as breeding, but decreasing further to 3 or 4 did not add any. Further results from the PCA and clustering analyses were very similar (see Fig. 1 and Table 1). As a result, we chose to keep all growth curves with at least 5 points.

Minimal number of points to constitute a growth curve	Method	Method details	nb curves	nb imputed values	nb imputed values (%)	PC1 %	PC2 %	PC3 %	nb clusters HCPC	Nb breeding adults, long term monitoring
	**1**	PCA on all parameters (11) + Clustering on PCA axes (old method)	2444	1875	6.90%	27.70%	18.20%	15.60%	3	
3	**2**	PCA on parameters with cor < 0.5 (7) + Clustering on PCA axes	2444	683	4.00%	29.80%	18.70%	16.00%	4	0
	**3**	PCA on all parameters (11) + Clustering on 4 parameters deduced from the 3 PCA axes	2231	0	0	NA	NA	NA	3	
	**1**	PCA on all parameters (11) + Clustering on PCA axes (old method)	2369	1428	5.50%	27.00%	18.40%	15.60%	3	
4	**2**	PCA on parameters with cor < 0.5 (7) + Clustering on PCA axes	2369	514	3.10%	29.00%	18.80%	16.40%	3	0
	**3**	PCA on all parameters (11) + Clustering on 4 parameters deduced from the 3 PCA axes	2231	0	0	NA	NA	NA	3	
	**1**	PCA on all parameters (11) + Clustering on PCA axes (old method)	2298	1031	4.10%	26.60%	18.70%	15.40%	3	
5	**2**	**PCA on parameters with cor < 0.5 (7) + Clustering on PCA axes**	**2298**	**357**	**2.20%**	**28.20%**	**18.70%**	**16.80%**	**3**	2
	**3**	PCA on all parameters (11) + Clustering on 4 parameters deduced from the 3 PCA axes	2231	0	0	NA	NA	NA	3	
	**1**	PCA on all parameters (11) + Clustering on PCA axes (old method)	2231	875	3.60%	26.30%	18.90%	15.30%	3	
6	**2**	PCA on parameters with cor < 0.5 (7) + Clustering on PCA axes	2231	275	1.80%	27.80%	18.60%	17.30%	3	1
	**3**	PCA on all parameters (11) + Clustering on 4 parameters deduced from the 3 PCA axes	2231	0	0	NA	NA	NA	3	
	**1**	PCA on all parameters (11) + Clustering on PCA axes (old method)	2121	765	3.30%	25.60%	19.70%	15.20%	3	
7	**2**	PCA on parameters with cor < 0.5 (7) + Clustering on PCA axes	2121	257	1.70%	27.00%	18.50%	18.20%	3	3
	**3**	PCA on all parameters (11) + Clustering on 4 parameters deduced from the 3 PCA axes	2121	0	0	NA	NA	NA	3	

Table 1: Sensitivity analysis of the clustering method. The first column corresponds to “Minimal number of points to constitute a growth curve”. We tested the

clustering method on different dataset of growth curves. The column “Method” corresponds to the 3 methods tested and the details are given in the column “Method details”. The “number of curves” vary with the minimal number of points to constitute a growth curve. Fewer curves are constituted with 6 or 7 points; therefore, the number of curves decreases. “Imputed values” were calculated with the ImputePCA function, for the methods 1 and 2. The 3rd method is a clustering ran on complete cases only. Therefore, the number of curves is lower than for methods 1 and 2. The % of explained variance for the first 3 axes (PC1, PC2, PC3) are then given for the method 1 and 2, as well as the “number of clusters” calculated with the unsupervised HCPC method. The “number of breeding adults” correspond to chicks followed during the growth that were found later in the long-term monitoring but with exactly 3, 4, 5, 6 or 7 points. This has been calculated to ensure that all chicks monitored for reproduction remain in our dataset.

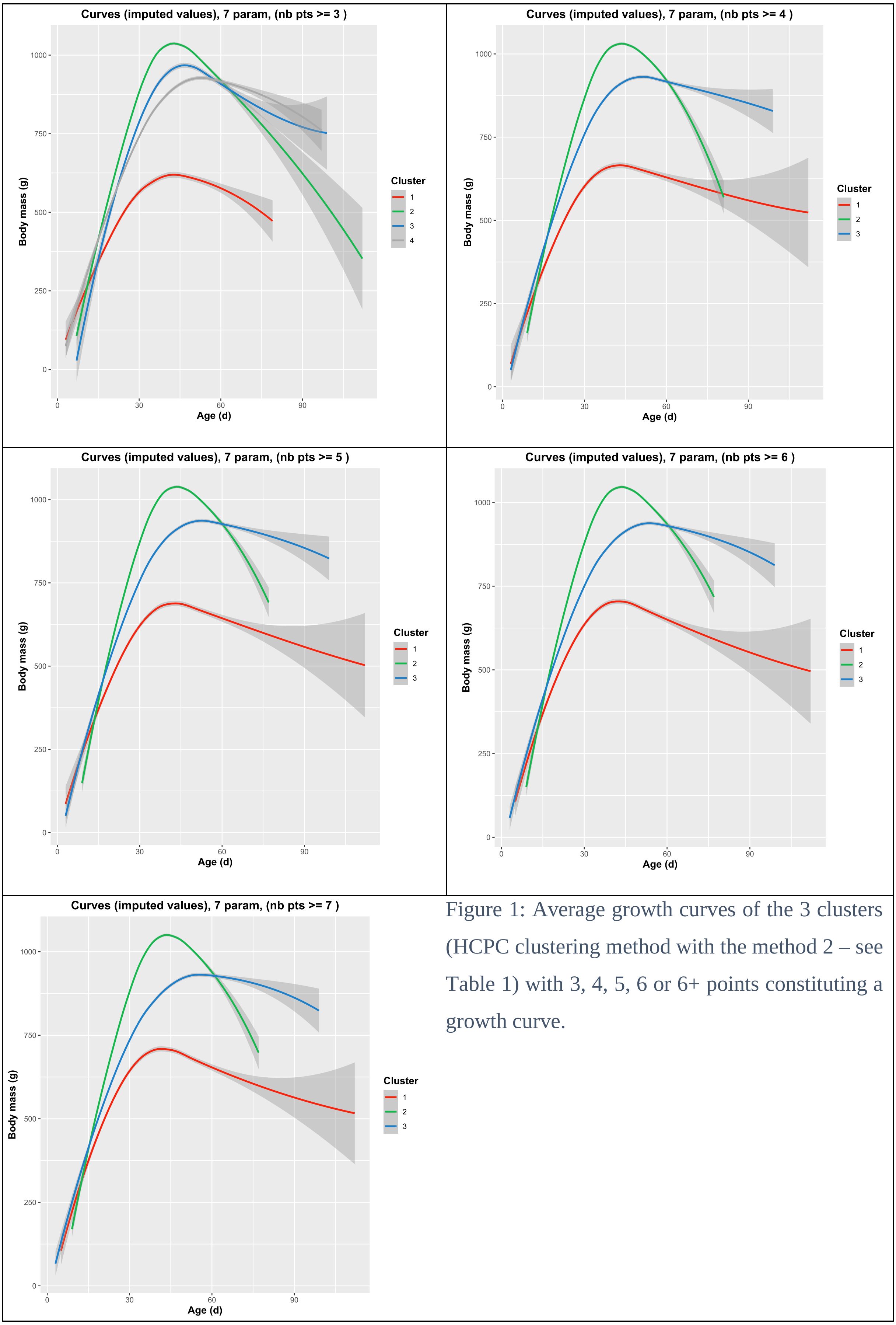

Figure 1: Average growth curves of the 3 clusters (HCPC clustering method with the method 2 – see Table 1) with 3, 4, 5, 6 or 6+ points constituting a growth curve.

2. *Clustering method*

After testing k-means and HCPC clustering methods, we chose the HCPC for its robustness on our dataset. Indeed, the number of clusters almost never changed regardless of the minimal number of points to keep in a curve (see Table 1 and Fig. 1), or the method used (1, 2, 3 in Table 1- either by clustering on PCA axes or directly on the growth parameters).

3. *PCA variables and Imputed values*

Another important point was to decide which variables to use in the PCA. Indeed, adding correlated variables would bias the first axes of the PCA towards those variables and then be more represented in our clustering. Further, adding variables also increases the number of missing values to impute, which could introduce uncertainty.

Some of the growth variables were highly correlated (cor >0.5 according to Cohen, 1988, 1992). We therefore compared our initial PCA with 11 variables with one in which only variables with low or moderate correlations (*i.e.* cor <0.5) were kept. Finally, we also compared the clustering results performed on these 2 PCA with clustering directly on 4 parameters (defined to represent the most variability in the curve based on the PCA).

1) The first method (in red in the Table 1) uses all PCA input variables (11 growth variables, Table 2). Some are highly correlated with each other (cor >0.5), notably fledging mass with peak mass (cor = 0.67), or age at peak mass with fledging age (cor = 0.62), or fledging mass and relative loss mass (cor= -0.77). This could artificially increase the weight given to those variables as they should load more on the first axes that are kept for the clustering afterwards. Besides, the number of impute values is higher (4.1% for 5 points), since fledging parameters (mass and age) were only known for chicks that have fledged and had to be estimated for all others.

2) The second method (in green in the Table 1) uses only the 7 variables (Table 2) with a correlation of less than 0.5 (moderate correlation according to Cohen, 1988, 1992), and avoids variables linked to fledging. This drastically reduces the number of imputed values in the PCA (2.2%).

3) The third method (in black in the Table 1) uses the PCA to define variables that have high and as independent as possible contribution on the first 3 axes of the PCA. This left us with a group of 4 variables (peak mass, guard duration, slope, irregularity,

see Table 2). The clustering method is then applied directly to the variables, and not to the PCA axes. This method means that we do not impute any data. Further, because we take fewer parameters into account, we might lose some important details.

When comparing the results of these 3 methods, we always found 3 clusters. The first two methods gave very similar results (78% similarity), while the third one gave slightly different growth curves (59% and 54% similarity with method 1 and method 2 respectively) (Fig. 2 and Table 3).

Table 2: Parameters used for the 3 methods

Parameter	Method 1	Method 2	Method 3
**Peak mass**	X	X	X
**Mass at fledging**	X		
**Relative Loss of Mass**	X	X	
**Delta AB**	X	X	
**Guard duration**	X	X	X
**Steepest slope**	X	X	X
**Duration of the steepest slope**	X		
**Start of the steepest slope**	X		
**Age at peak mass**	X	X	
**Age at fledging**	X		
**Irregularity index**	X	X	X

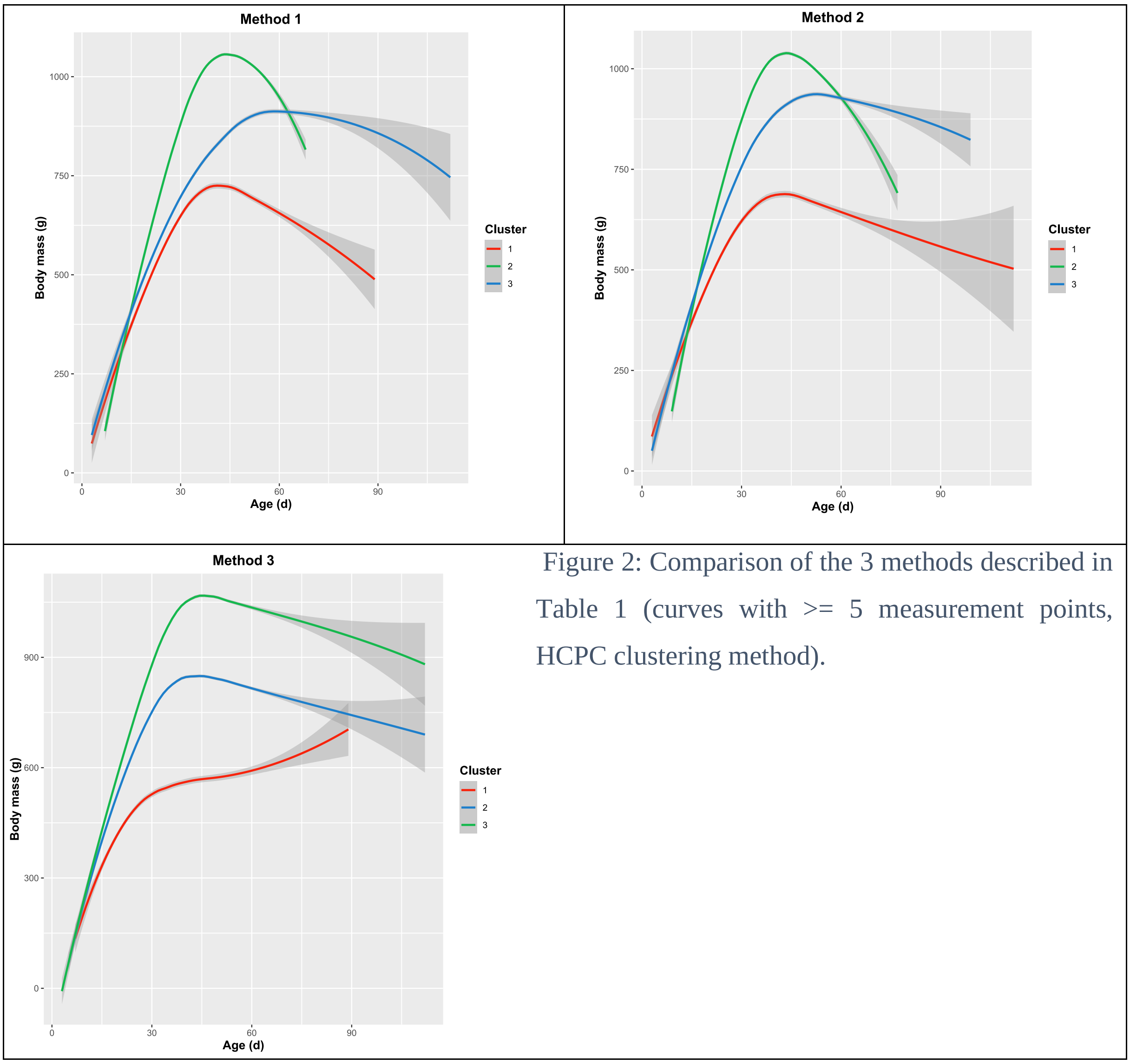

Figure 2: Comparison of the 3 methods described in Table 1 (curves with >= 5 measurement points, HCPC clustering method).

Table 3: Similarity of clusters between the 3 methods

	% Similarity between clusters
**Method 1 vs Method 2**	78 %
**Method 1 vs Method 3**	59 %
**Method 2 vs Method 3**	54 %

In conclusion, our sensitivity analysis proved our results were quite robust. Using 3 different methods on 5 different datasets (depending on the number of minimum points to consider an individual), we found 3 clusters in 14 out of 15 cases. We chose the second method which seemed the best compromise between keeping as many variables as possible to be the most informative and avoiding bias due to either variables being too correlated or imputation of values. This method was applied on growth curves comprising at least 5 points, again as the

best compromise between considering as many individuals as possible and having enough data to accurately estimate the different parameters.

4. Final results: PCA and clustering

Here are the PCA and clustering results (Fig. 3, Table 4 and Fig. 4).

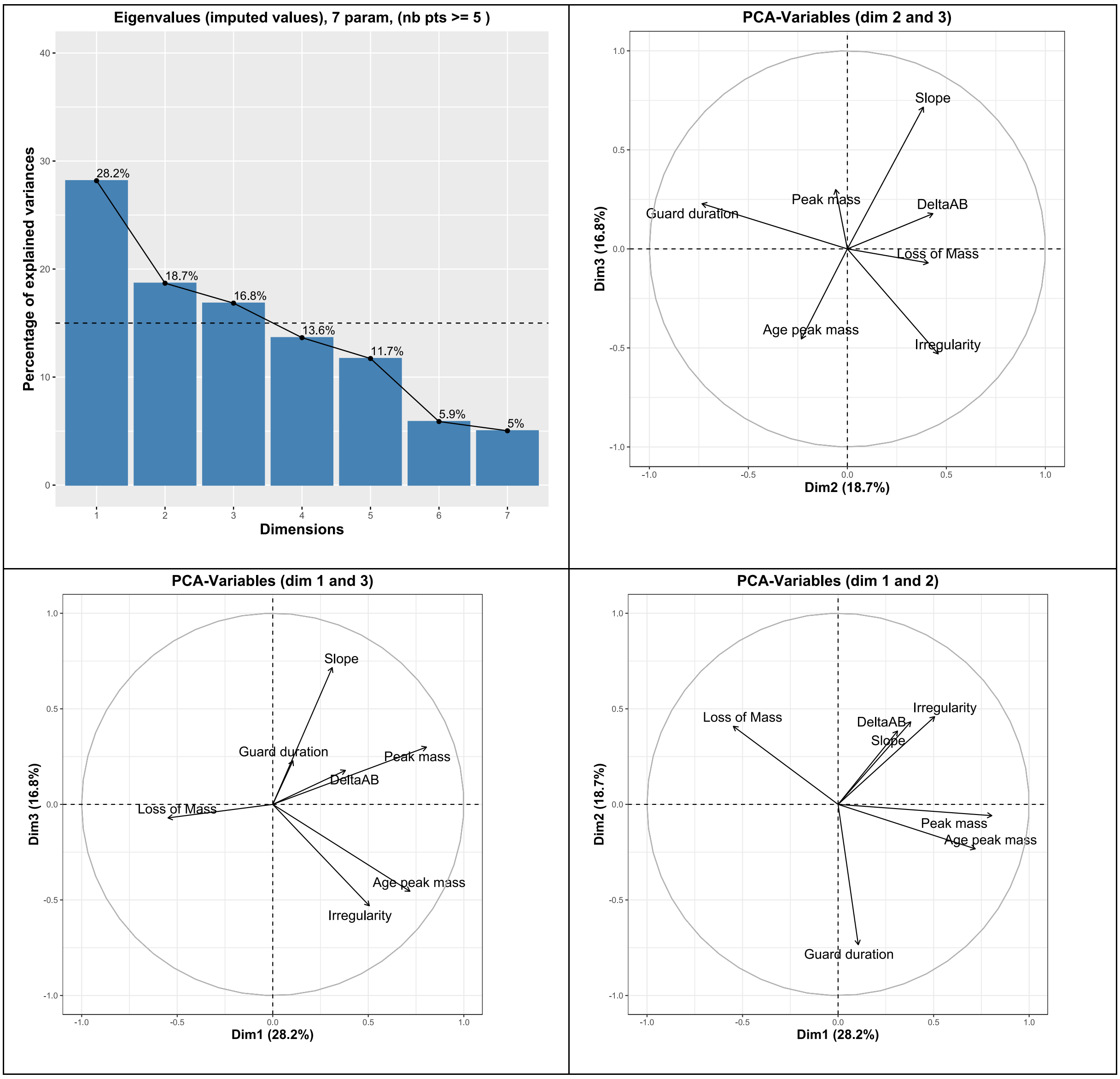

Figure 3: PCA results (HCPC, method 2, curves constituted with at least 5 points)

Table 4: Contribution (%) of the 7 variables on the first 3 PCA axes.

	Dim 1	Dim 2	Dim 3
**Peak body mass**	32.8	0.3	7.6
**Guard duration**	0.6	41.2	4.4
**Irregularity**	13.0	16.1	23.9
**DeltaAB**	7.3	14.3	2.7
**Relative loss of mass**	15.2	12.8	0.4
**Slope**	4.9	11.3	43.4
**Age at peak mass**	26.1	4.1	17.5

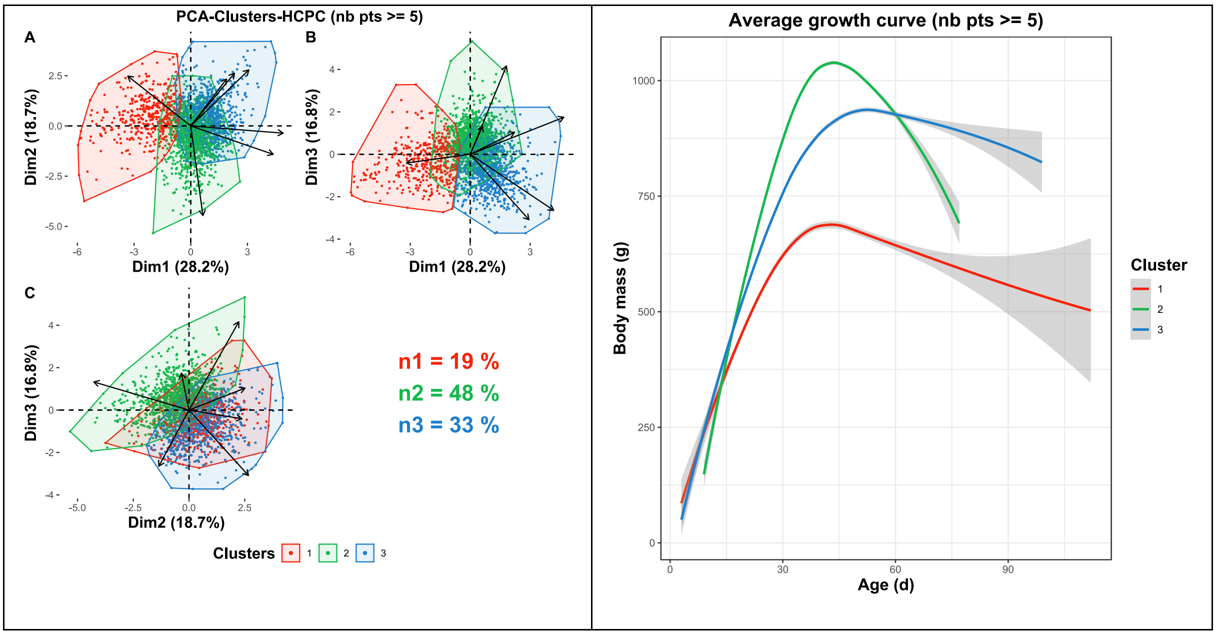

Figure 4: Clustering results on the first 3 PCA axes and the growth curves corresponding

Appendix 3: Capture Mark Recapture Model

Survival rates were estimated using a Capture Mark Recapture model (CMR). The analysis is based on capture–recapture histories of birds caught for the first-time during fledging and recaptured in subsequent years as juveniles and then breeders. The data covers the period from 1995 to 2022. The construction and choice of model are detailed in the appendix. CMR models estimate survival probabilities (φ, the probability that a penguin will survive between year *t* and year *t+1*) and capture probabilities (P, the probability that a penguin alive at year *t* is caught during the breeding season of year *t*). In our case, a capture corresponds to a penguin that was recorded at the nest during the breeding season or by the automatic penguin monitoring system (i.e. transponder readers situated at two entry points of the colony).

To ensure model assumptions were validated, we ran goodness-of-fit (GOF) procedures for each growth phenotypes using U-care (Choquet et al., 2005; Gimenez et al., 2018). Tests 3.SR revealed transience dynamics for fast and slow phenotypes, which often results from the mix of juveniles and adults (large difference in survival rates) within the same population (Appendix 3A). Consistently with our life cycle (Figure 2 in the manuscript), we thus included 5 age-classes in our model (denoted by *a*): S0, S1, S2, S3 and Sa (Pradel et al., 1997). Tests 2.CT revealed trap-dependence for the fast phenotype (Appendix 3A). Trap-dependence is common in sea-bird capture-recapture data, because of their loyalty to their breeding site: an individual captured in year *t* is more likely to be recaptured in year *t+1* than a bird not seen in *t* (*i.e.* "trap-happiness"; Jenouvrier et al., 2005, 2009). To deal with trap-dependence (denoted by *m*), we ran a multievent CMR model with E-SURGE software (Pradel, 2005).

The CJS model fitted the data (P=0.13) for the "light" chicks. The transient models (φ_a, P) fitted the data (P = 0.04) for the "slow" chicks. The model coupled with transience and trap dependance (φ_a, P_m) fitted the data for the "fast" chicks (P = 0.96). The umbrella (*i.e.* most general) model was thus $\varphi_{a.g.c}$, $P_{a.g.m}$ and its construction is detailed in the Appendix 3B (see also Pradel & Sanz-Aguilar, 2012). From the umbrella model, a backward model selection was run (Appendix 3C), based on the QAICc. Quasi Akaike's information criterion corrected for small sample size (QAICc; Anderson et al., 1994; Lebreton et al., 1992) is used for model comparison for overdispersed count data. The selected model was the one with the lowest QAICc, (i.e. the most parsimonious model in terms of the number of parameters and deviance). CMR outputs were given with the 95% CI given by E-SURGE (see Choquet & Nogue, 2011 for more details).

Appendix 3A: Tests GOF. Empty boxes refer to cases when there were not sufficient data to compute GOF test, grey boxes refer to cases when the test is not required in the procedure and significant tests appear in bold.

	Overall CJS	Test												GOF test for the umbrella model with…								
Growth cluster		3SR (transience)			3SM			2CT (trap)			2CL			Trap dependance			Transients			Transience + Trap dependence		
	P	df	χ^2	P	df	χ^2	P	df	χ^2	P	df	χ^2	P	df	χ^2	P	df	χ^2	P	df	χ^2	P
1 (LIGHT)	0.134	**Data fit the CJS model**																				
2 (FAST)	**0.00**	18	497.6	**0.00**	7	4.3	0.742	17	117.6	**0.00**	7	1.9	0.967	32	503.8	**0.00**	31	123.8	**0.00**	14	6.2	0.961
3 (SLOW)	**0.00**	18	305.2	**0.00**	5	6.35	0.274	8	17.1	0.03							13	23.5	0.04			

Appendix 3B: Implementation of the CMR model with transience and trap-dependence in E-Surge

To implement the model taking into account both the effects of trap dependance on detection probabilities and transience on survival probabilities, we followed the method introduced by (Pradel & Sanz-Aguilar, 2012).

We implemented the model as a multievent model in program E-SURGE.

The individual states (s=3) considered are:

- **A**, previously seen
- **U**, not previously seen
- **D**, dead

The possible events (e=2) are:

- **0**, not recaptured
- **1**, captured or recaptured

The symbols for parameters are:

- **ϕ**, survival probability
- **p**, capture probability

The matrix of initial state is assessed at the time of initial release (first released after marking). Therefore, all individuals, captured as fledglings, are necessarily 'previously seen'. The proportion of 'previously seen' is 1.

Initial State probabilities:

A	U
1	0

The transition probabilities is separated in two processes: the survival process (S), which takes place between times t+ and t+1- (just before the capture session t+1) and the trap awareness process (P), which takes place between t+1- and t+1+. These two processes are defined as step 1 and step 2 in E-Surge and the matrixes are described below.

Transition probabilities, step 1 (S):

	**A_{t+1^-}**	**U_{t+1^-}**	**D_{t+1^-}**
A_t	ϕ	0	1-ϕ
U_t	0	ϕ	1-ϕ
D_t	0	0	1

Transition probabilities, step 2 (P):

	$\mathbf{A}_{t+1}$	$\mathbf{U}_{t+1}$	$\mathbf{D}_{t+1}$
$\mathbf{A}_{t+1}^{-}$	p’	1-p’	0
$\mathbf{U}_{t+1}^{-}$	p	1-p	0
$\mathbf{D}_{t+1}^{-}$	0	0	1

If an animal is trap-aware at t+, that means that it has just been captured (conventional code ‘1’). If it is trap unaware or dead, it has not been captured during this session (conventional code ‘0’). This is summarized in the Event probabilities matrix (E) with states in row and events in column.

Event probabilities:

	**0**	**1**
$\mathbf{A}_{t}$	0	1
$\mathbf{U}_{t}$	1	0
$\mathbf{D}_{t}$	1	0

Appendix 3C: Capture-Mark-Recapture, model selection

Model name	Tested parameters	K	Deviance	QAICc	Delta
**Umbrella 1**	$\Phi_{a\, x\, g + c + t}$ $P_{a\, x\, g + m}$	83	2276	2447	15
**2**	$\Phi_{a\, x\, g + c + t}$ $P_{a + g + m}$	75	2281	2436	4
**3**	$\mathbf{\Phi_{a\, x\, g + c + t}}$ $\mathbf{P_{a + m}}$	**73**	**2282**	**2432**	**0**
**4**	$\Phi_{a\, x\, g + c + t}$ $P_{g + m}$	71	2305	2451	19
**5**	$\Phi_{a\, x\, g + c + t}$ P_{m}	69	2306	2448	16
**Umbrella 2**	$\Phi_{a\, x\, g + c + t}$ $P_{a + m}$	73	2282	2432	19
**7**	$\Phi_{a + g + c + t}$ $P_{a + m}$	61	2320	2445	32
**8**	$\mathbf{\Phi_{a + g + c}}$ $\mathbf{P_{a + m}}$	**38**	**2335**	**2413**	**0**
9	$\Phi_{a + g}$ $P_{a + m}$	13	2420	2446	33
10	$\Phi_{a + c}$ $P_{a + m}$	36	2350	2423	10
11	Φ_{a} $P_{a + m}$	11	2429	2451	38
**Neutral**	Φ. P.	2	4223	4227	1814

Quasi Akaike's information criterion, corrected for small sample size (QAICc; (Lebreton et al., 1992) is used for model comparison. Delta is the difference of QAICc between the specified model and the best one (i.e. Delta = 0 for the best model) in each table. K refers

to the rank and Deviance to the deviance of the model. An effect of growth cluster on survival or capture rate is denoted by g, an effect of cohort is denoted by c, the letter m denotes apparent 'trap happiness' on recapture rates, and a transient effect on adult survival is denoted by a (see 'Material and methods' for more details). The model in red is the chosen model, with the lowest QAICc.

CMR model selection indicated that the most parsimonious model was $\Phi_{a + g + c}$ $P_{a + m}$ including age (5 age classes; S0, S1, S2, S3, Sa), growth cluster (fast, slow and light) and cohort effects on survival probabilities and an effect of trap-dependence and age (5 age classes; S0, S1, S2, S3, Sa) on capture probabilities. No interaction was found between growth clusters and age, indicating a constant effect of the growth cluster on survival into adulthood.

Appendix 3D: Capture-Mark-Recapture, capture probabilities

	S0	S1	S2	S3	Sa
**Capture probabilities**	0,76 ± 0,07	0,97 ± 0,01	0,94 ± 0,03	0,95 ± 0,03	0,93 ± 0,03

Appendix 4: Markov chain formulation of the life cycle

The transition matrix for the absorbing Markov chain is given as:

$$P = \begin{pmatrix} U & 0 \\ m^{\top} & 1 \end{pmatrix}$$

where **U** contains probabilities of transition and survival for living individuals and $m^{\top}$ includes the mortality probabilities: $m^{\top} = 1^{\top} - 1^{\top}U$.

Based on defined breeding stages and vital rates, U is given by:

$$U = \begin{pmatrix} 0 & 0 & 0 & 0 & 0 \\ h\,f\,S_0 & 0 & 0 & 0 & 0 \\ 0 & S_1(1-P_2) & 0 & 0 & 0 \\ 0 & 0 & S_2(1-P_3) & S_3(1-P_4) & 0 \\ 0 & S_1P_2 & S_2P_3 & S_3P_4 & S_a \end{pmatrix}$$

From this transition matrix, the fundamental matrix **N** was deduced as $N = (I - U)^{-1}$ and occupation times in each of the states were then derived from **N** (see Caswell, 2001; Van Daalen & Caswell, 2017 for calculations).

Appendix 5: Growth parameters for the three clusters (Comparison between the three clusters performed using the ANOVA statistical test, followed by the Tukey post-HOC test. Significance codes: 0.0001 **, 0.001 ***, 0.01 **, 0.05 *, 0.1 ns) with associated values (+/- SD).**

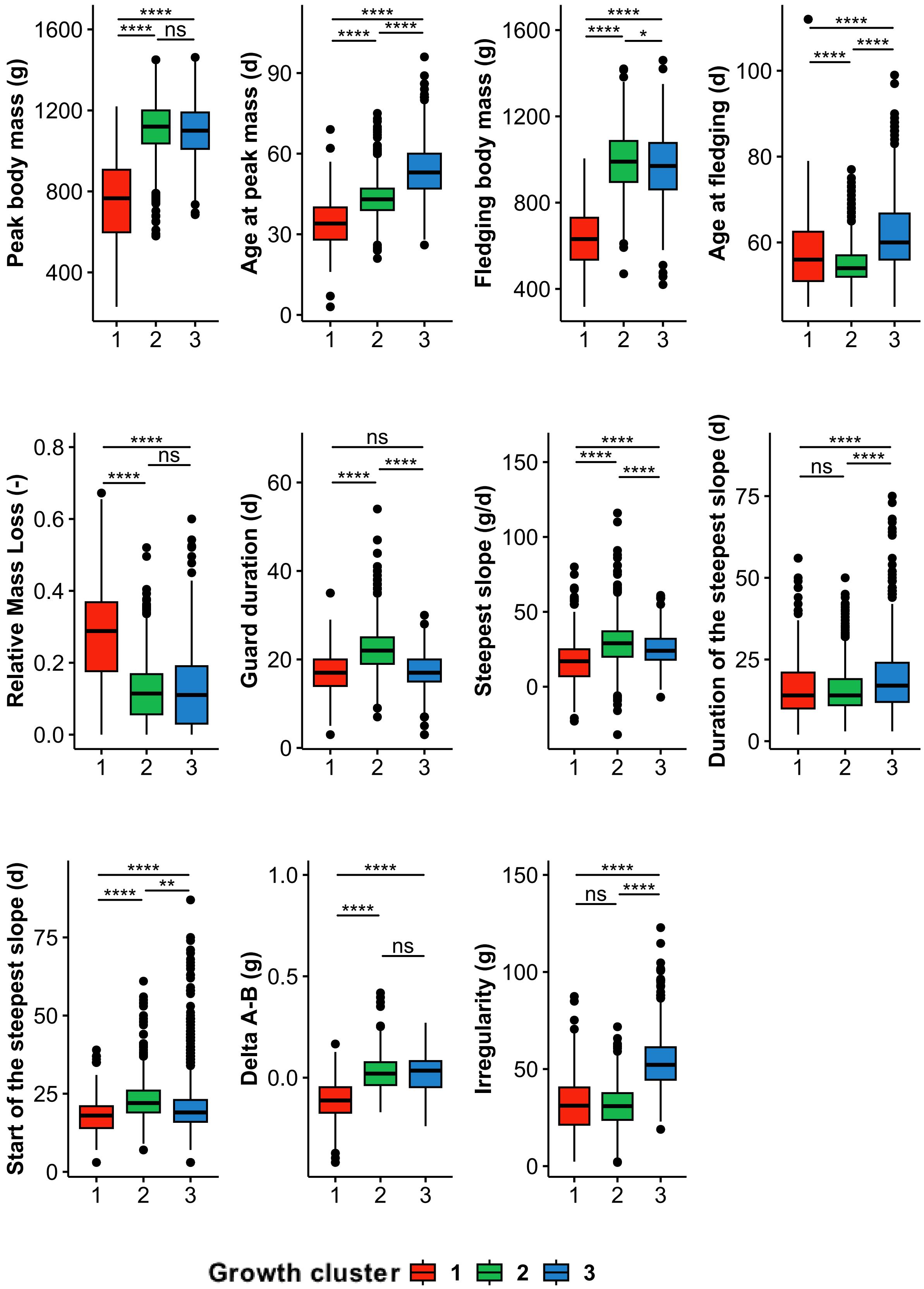
Peak body mass (g)
Age at peak mass (d)
Fledging body mass (g)
Age at fledging (d)
Relative Mass Loss (-)
Guard duration (d)
Steepest slope (g/d)
Duration of the steepest slope (d)
Start of the steepest slope (d)
Delta A-B (g)
Irregularity (g)
Growth cluster 1 2 3

Growth parameter	***Light***	***Fast***	***Slow***
Peak body mass (g)	739 ± 216^{a}	1114 ± 119^{b}	1100 ± 136^{b}
Age at peak body mass (d)	34 ± 9^{a}	44 ± 6^{b}	54 ± 10^{c}
Fledging body mass (g)	622 ± 136^{a}	990 ± 133^{b}	966 ± 163^{b}
Age at fledging (d)	58 ± 7^{a}	55 ± 4^{b}	62 ± 7^{c}
Relative loss of mass (-)	0.28 ± 0.14^{a}	0.11 ± 0.08^{b}	0.12 ± 0.12^{b}
Guard duration (d)	17 ± 4^{a}	23 ± 4^{b}	17 ± 4^{a}
Steepest slope (g/d)	18 ± 13^{a}	28 ± 12^{b}	25 ± 10^{c}
Duration of the steepest slope (d)	16 ± 7^{a}	16 ± 6^{a}	20 ± 7^{b}
Start of the steepest slope (d)	18 ± 4^{a}	23 ± 4^{b}	22 ± 6^{c}
Relative delta (A/B) (-)	-0.11 ± 0.09^{a}	0.02 ± 0.08^{b}	0.03 ± 0.10^{b}
Irregularity index (g)	32 ± 14^{a}	31 ± 10^{a}	54 ± 12^{b}

Appendix 6: Matrixes U, F, N. U gives the probabilities of transition and survival for living individuals, F gives the fertility probabilities and N is the fundamental matrix.

$$U_{Fast} = \begin{pmatrix} 0 & 0 & 0 & 0 & 0 \\ 0.153 & 0 & 0 & 0 & 0 \\ 0 & 0.371 & 0 & 0 & 0 \\ 0 & 0 & 0.415 & 0 & 0 \\ 0 & 0.329 & 0.415 & 0.780 & 0.900 \end{pmatrix}$$

$$U_{Slow} = \begin{pmatrix} 0 & 0 & 0 & 0 & 0 \\ 0.133 & 0 & 0 & 0 & 0 \\ 0 & 0.469 & 0 & 0 & 0 \\ 0 & 0 & 0.376 & 0 & 0 \\ 0 & 0.191 & 0.424 & 0.910 & 0.880 \end{pmatrix}$$

$$U_{Light} = \begin{pmatrix} 0 & 0 & 0 & 0 & 0 \\ 0.025 & 0 & 0 & 0 & 0 \\ 0 & 0.155 & 0 & 0 & 0 \\ 0 & 0 & 0.650 & 0 & 0 \\ 0 & 0.315 & 0 & 0.820 & 0.770 \end{pmatrix}$$

$$F_{Fast} = \begin{pmatrix} 0 & 0.434 & 0.548 & 1.030 & 1.189 \\ 0 & 0 & 0 & 0 & 0 \\ 0 & 0 & 0 & 0 & 0 \\ 0 & 0 & 0 & 0 & 0 \\ 0 & 0 & 0 & 0 & 0 \end{pmatrix}$$

$$F_{Slow} = \begin{pmatrix} 0 & 0.249 & 0.551 & 1.183 & 1.144 \\ 0 & 0 & 0 & 0 & 0 \\ 0 & 0 & 0 & 0 & 0 \\ 0 & 0 & 0 & 0 & 0 \\ 0 & 0 & 0 & 0 & 0 \end{pmatrix}$$

$$F_{Light} = \begin{pmatrix} 0 & 0.353 & 0 & 0.918 & 0.862 \\ 0 & 0 & 0 & 0 & 0 \\ 0 & 0 & 0 & 0 & 0 \\ 0 & 0 & 0 & 0 & 0 \\ 0 & 0 & 0 & 0 & 0 \end{pmatrix}$$

$$N_{Fast} = \begin{pmatrix} 1 & 0 & 0 & 0 & 0 \\ 0.153 & 1 & 0 & 0 & 0 \\ 0.057 & 0.371 & 1 & 0 & 0 \\ 0.024 & 0.154 & 0.415 & 1 & 0 \\ 0.922 & 6.031 & 7.387 & 7.800 & 10.0 \end{pmatrix}$$

$$N_{Slow} = \begin{pmatrix} 1 & 0 & 0 & 0 & 0 \\ 0.133 & 1 & 0 & 0 & 0 \\ 0.062 & 0.469 & 1 & 0 & 0 \\ 0.023 & 0.176 & 0.376 & 1 & 0 \\ 0.608 & 4.587 & 6.385 & 7.583 & 8.333 \end{pmatrix}$$

$$N_{Light} = \begin{pmatrix} 1 & 0 & 0 & 0 & 0 \\ 0.024 & 1 & 0 & 0 & 0 \\ 0.004 & 0.155 & 1 & 0 & 0 \\ 0.003 & 0.100 & 0.650 & 1 & 0 \\ 0.043 & 1.728 & 2.317 & 3.565 & 4.348 \end{pmatrix}$$

Appendix 7: LRO in terms of fledglings

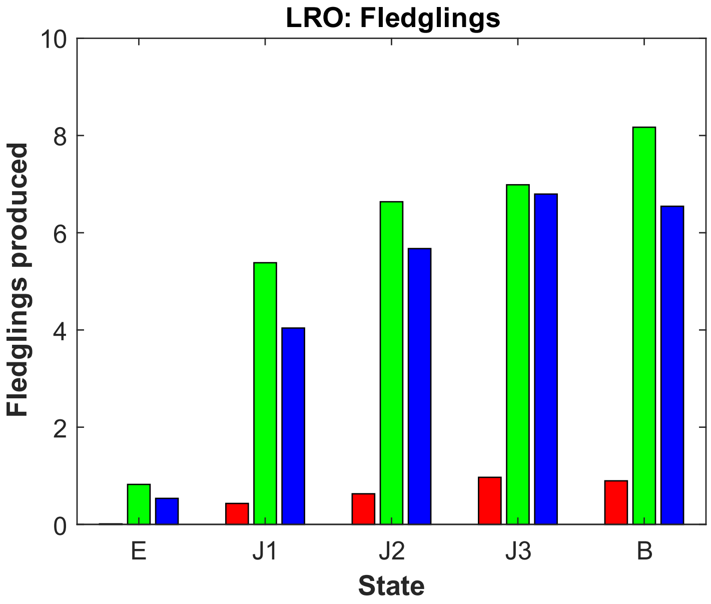

Fast individuals were the most efficient producing fledglings and light ones were less productive, regardless of the initial state. On average, a fast breeder will produce 8.2 fledglings, i.e. 1.2 times more as a slow breeder (6.5) and 9 times more as a light breeder (0.9). This is partly due to the fact that three different demographic models were considered here, for each growth cluster. Thus, a fast egg necessarily produced a fast egg, with the same fledging success as its parent, which greatly handicapped the light individuals with the lowest fledging success.

REFERENCES

Anderson, D. R., Burnham, K. P., & White, G. C. (1994). AIC Model Selection in Overdispersed Capture-Recapture Data. *Ecology*, *75*(6), 1780–1793. https://doi.org/10.2307/1939637

Caswell, H. (2001). *Matrix Population Models—Construction, analysis, and interpretation*. Sinauer Associates, Incorporated.

Choquet, R., & Nogue, E. (2011). *E-SURGE 1.8 user's manual*. http://ftp.cefe.cnrs.fr/biom/soft-cr/

Choquet, R., Reboulet, A., Lebreton, J.-D., Gimenez, O., & Pradel, R. (2005). *U-CARE-22-users-manual-Utilities-CApture-REcapture.pdf*. https://www.researchgate.net/profile/Jean-Dominique-Lebreton/publication/273138843_U-CARE_22_user%27s_manual_Utilities-CApture-REcapture/links/550741610cf2d7a281237b4c/U-CARE-22-users-manual-Utilities-CApture-REcapture.pdf

Cohen, J. (1988). *Statistical power analysis for the behavioral sciences* (2nd ed). L. Erlbaum Associates.

Cohen, J. (1992). Quantitative methods in psychology. *Psychological Bulletin*, *112*(1), 155–159.

Gimenez, O., Lebreton, J., Choquet, R., & Pradel, R. (2018). R2ucare: An R package to perform goodness-of-fit tests for capture–recapture models. *Methods in Ecology and Evolution*, *9*(7), 1749–1754. https://doi.org/10.1111/2041-210X.13014

Jenouvrier, S., Barbraud, C., & Weimerskirch, H. (2005). Long-Term Contrasted Responses to Climate of Two Antarctic Seabird Species. *Ecology*, *86*(11), 2889–2903. https://doi.org/10.1890/05-0514

Jenouvrier, S., Thibault, J., Viallefont, A., Vidal, P., Ristow, D., Mougin, J., Brichetti, P., Borg, J. J., & Bretagnolle, V. (2009). Global climate patterns explain range-wide synchronicity in survival of a migratory seabird. *Global Change Biology*, *15*(1), 268–279. https://doi.org/10.1111/j.1365-2486.2008.01715.x

Lebreton, J.-D., Burnham, K. P., Clobert, J., & Anderson, D. R. (1992). Modeling Survival and Testing Biological Hypotheses Using Marked Animals: A Unified Approach with Case Studies. *Ecological Monographs*, *62*(1), 67–118. https://doi.org/10.2307/2937171

Pradel, R. (2005). Multievent: An Extension of Multistate Capture–Recapture Models to Uncertain States. *Biometrics*, *61*(2), 442–447. https://doi.org/10.1111/j.1541-0420.2005.00318.x

Pradel, R., Hines, J. E., Lebreton, J.-D., & Nichols, J. D. (1997). Capture-Recapture Survival Models Taking Account of Transients. *Biometrics*, *53*(1), 60. https://doi.org/10.2307/2533097

Pradel, R., & Sanz-Aguilar, A. (2012). Modeling Trap-Awareness and Related Phenomena in Capture-Recapture Studies. *PLoS ONE*, *7*(3), e32666. https://doi.org/10.1371/journal.pone.0032666

Simonoff, J. S. (1996). *Smoothing Methods in Statistics* (Springer).

Van Daalen, S. F., & Caswell, H. (2017). Lifetime reproductive output: Individual stochasticity, variance, and sensitivity analysis. *Theoretical Ecology*, *10*(3), 355–374. https://doi.org/10.1007/s12080-017-0335-2
